# First Direct Observation of Equilibrium Involving Cl Atoms: Cl + C_2_H_4_ ⇔ ClCH_2_CH_2_ by VUV Monitoring

**DOI:** 10.1021/acs.jpca.5c05430

**Published:** 2025-10-08

**Authors:** Mark A. Blitz, Thomas Henry Speak, Paul W. Seakins

**Affiliations:** † School of Chemistry, 4468 University of Leeds, Leeds LS2 9JT, U.K.; ‡ NCAS, University of Leeds, Leeds LS2 9JT, U.K.; § University of British Columbia, Vancouver, Vancouver V6T 1Z1, BC, Canada

## Abstract

The kinetics between Cl and ethylene, R1, have been determined between 298 and 822 K in time-resolved experiments, where the Cl atoms were monitored for the first time via laser-induced fluorescence at 118.877 nm. A key advantage of this method of Cl detection is that there is limited absorption of 118 nm radiation by oxygen, and we report the first Cl reactivity measurements. The kinetics of Cl + C_2_H_4_ at room temperature are simple association, *k*_1a([*M*])_, and have been used in a master equation analysis (via the MESMER application) to show consistency with most of the literature. Between 393 and 490 K, the kinetics exhibited equilibrium behavior: Cl + C_2_H_4_ ⇔ Cl–C_2_H_4_ (*k*_1a([*M*])_,*k*_–1a([*M*])_). These forward and reverse rate coefficients have been used in van’t Hoff and reaction rate theory (MESMER) analysis to determine the enthalpy of reaction, Δ_r_ *H*_R1a_^o^. This analysis yields a Δ_r_ *H*_0,R1a_^o^ equal to −74.1 ± 0.6 kJ mol^–1^. *Ab initio* structure calculations provided input values for MESMER analysis of the equilibrium data. The range of *ab initio* calculations carried out returned consistent values for Δ_r_ *H*_0,R1a_^o^, but the values are consistently more exothermic than the experimental value. Via comparison between theory and experiment, it is estimated that these *ab initio* calculations are good to ∼4 kJ mol^–1^. Above 500 K, the removal kinetics are dominated by abstraction: Cl + C_2_H_4_ → HCl + C_2_H_3_ (*k*_1b_). This reaction occurs on an endothermic potential surface, where the energy of the transition state is below that of the products. Analysis of this kinetic data and the literature highlights that the location of the transition state along the reaction coordinate varies with temperature, becoming more reagent-like with increased temperature.

## Graphical abstract

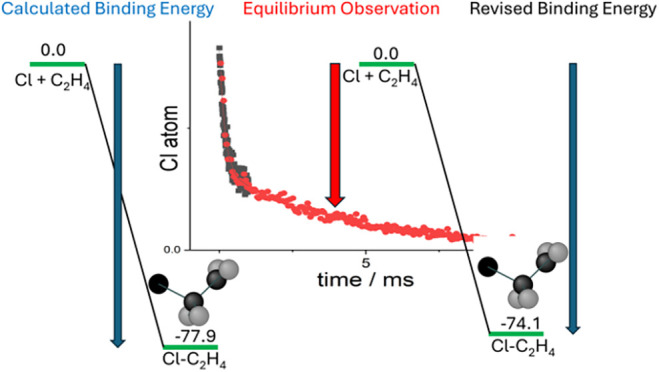

## 1 Introduction

Cl atoms play important roles in a variety of complex chemical processes, ranging from atmospheric chemistry to plasma processing and combustion. Accurate measurement of rate coefficients for Cl atom reactions is vital for modeling such systems. OH radicals are the primary atmospheric oxidants,^1^ but observation of the Cl precursor ClNO_2_ over the continental USA^2^ has raised the potential that Cl-induced chemistry may be much more widespread than the marine boundary layer.^3^ The atmospheric impact of Cl has been best quantified in the GEOS-Chem global 3-D model by Wang et al.,^4^ where the global [Cl] is estimated at 620 cm^–3^. Reactivity, *k*_*X*_, is defined as the pseudo-first-order loss of a radical species X (e.g., OH or Cl), *k*_*X*_ = ∑_*i*_ *k*_*i*_[*I*], where *k*_i_ is the bimolecular rate coefficient for the reaction of X with atmospheric species and *I* is the atmospheric species, typically a volatile organic compound, VOC. Reactivity is an important component in constraining radical budgets.^5^ To the best of our knowledge, there have been no direct Cl reactivity measurements; partially, we suspect due to the difficulty in determining pseudo-first order losses of Cl atoms under atmospheric conditions.

Historically, direct Cl atom kinetic studies have been performed in both flow-tube and laser flash photolysis systems, with Cl atom concentrations being followed by resonance fluorescence using resonance lamps (vacuum ultraviolet—VUV transitions in the region 133–139 nm). An exception to the resonance lamp studies for fluorescence detection of Cl is the use of degenerate four-wave mixing to generate light at 134.724 nm by the group of Matsumi and co-workers, e.g., Iwasaki et al., for a study on the reaction of Cl with ethylene.^6^

For saturated organic compounds, reactions proceed exclusively via abstraction, and the strong and selective IR absorption of HCl has been used to follow kinetics and extract overall branching ratios, e.g., Pilgrim and Taatjes.^7^ Selective deuteration allows for a determination of branching ratios from HCl production, e.g., Choi et al.^8^ In addition to the direct measurements, there has been a plethora of relative rate studies. Direct studies are essential to provide independent rate coefficients to put these relative studies on an absolute basis.

The technique for Cl atom detection described in this work has several important advantages over the fluorescence studies performed to date, most of which have utilized a microwave-driven resonance lamp to generate the excitation light. In this work, excitation (4s ^2^D_3/2_ ← 3p ^2^P_3/2_) occurs at 118.877 nm, whereas previous studies have used the transitions between 133 and 139 nm. 117.877 nm is in a ‘trough’ in the E transition of oxygen^9^ and even at atmospheric concentrations of O_2_ (∼5 × 10^18^ molecules cm^–3^) and a 10 cm path length (typical for kinetic measurements), ∼5% of the light is transmitted. In contrast, 5% transmission occurs at ∼0.01 atm of air at ∼134.7 nm over the same path length, limiting the applicability of conventional resonance fluorescence (RF) at ∼133–139 nm to study Cl in complex environments and precluding its use in atmospheric reactivity measurements. Unlike resonance lamps, which deliver a range of wavelengths (several Cl lines from different transitions plus emissions from any impurities), the laser-based method delivers a single probe wavelength, enhancing the specificity of the method. The frequency tripling method described in this work only requires the generation of a single probe wavelength, in contrast to the two UV systems required for four-wave mixing.^10^

The reaction of Cl with ethylene (ethene, C_2_H_4_) is a good system to test our Cl detection scheme. The reaction can occur via two channels; at low temperatures, addition ([Disp-formula R1a]) is the dominant process; however, as temperature increases, the abstraction channel becomes increasingly important:

R1a, R-1a $$\mathrm{Cl}+C_{2}H_{4}\overset{k_{1a\left([M]\right),}\;k_{-1a\left([M]\right)}\;}{↔}{\mathrm{ClC}}_{2}{\mathrm{CH}}_{4}$$

R1b $$\mathrm{Cl}+C_{2}H_{4}\overset{k_{1b}\;}{\to }\mathrm{HCl}+C_{2}H_{3}$$

The forward and reverse rate coefficients, *k*_1a([*M*])_ and *k*_–1a([M)_, are a function of [*M*], the total gas density. The lifetime of the chloroethyl radical becomes increasingly shorter as the temperature increases. At temperatures where the rates of the forward and reverse reactions become comparable, [Cl] approaches an equilibrium concentration, and *k*_1a([*M*])_ and *k*_–1a([*M*)_ can be extracted from the Cl atom traces, allowing for a direct determination of *K*_c,1a_ and Δ_r_ *H* ^o^ for reaction 1a. At high temperatures, abstraction dominates ([Disp-formula R1b]).

Reaction 1a has been studied (mostly at room temperature) by several groups using a variety of techniques, including Wallington et al.^11^ (relative rate, 100–3000 Torr), Kaiser and Wallington^12^ (relative rate, 0.2–100 Torr), Glowacki et al.^13^ (relative rate, ∼10–760 Torr), Stutz et al.^14^ (fast flow and RF and RR, ∼1 Torr), Iwasaki et al.^6^ (flash photolysis, four-wave mixing monitoring of Cl, 2–100 Torr), and Enghoff et al.^15^ (relative rate, 760 Torr). Pilgrim and Taatjes^16^ have studied reaction 1b via time-resolved observation of HCl product, and Knyazev et al.^17^ have examined the decomposition of CH_2_CH_2_Cl. There has been one *ab initio* calculation that calculates a well depth for the ClC_2_H_4_ adduct in the range 70–80 kJ mol^–1^.^18^ The lack of *ab initio* calculations on Cl compared to species containing just C, H, and O is related to the accuracy, where there is an unknown uncertainty associated with Cl. The thermodynamic parameters for the adduct measured from direct observation of equilibrium can then be compared with calculations to gauge the accuracy of the calculations.

In this paper, we detail the methodology used for Cl laser-induced fluorescence detection at 118.877 nm and report measurements of *k*_1_ over a range of conditions, allowing for a determination of Δ_f_ *H*(Cl–C_2_H_4_). We support our experimental determinations with calculations on Cl + C_2_H_4_ to provide further insight into [Disp-formula R1a] and R1b. Additionally, we present the first direct measurements of Cl reactivity from air sampled from outside the School of Chemistry in Leeds.

## 2 Methods

### 2.1 Experimental Section

The basic flash-photolysis—laser-induced fluorescence (LIF)—has been described before.^19–21^ It is the laser detection method used to monitor Cl atoms that is new. A multiaxis reaction cell admitted the gas flow containing the Cl atom precursor, ethylene, and the buffer gas (Ar, He, and N_2_). The gases were individually delivered by calibrated mass flow controllers that passed into a mixing manifold before entering the reaction cell. On one axis, a 266 nm laser (YAG, fourth harmonic, Quantel Q-Smart) photolyzed oxalyl chloride to form Cl atoms:

P1 $${\left(\mathrm{ClCO}\right)}_{2}+266\;\mathrm{nm}\to \mathrm{Cl}+\mathrm{CO}+\mathrm{Cl}\left(\mathrm{CO}\right)\to \mathrm{Cl}+\mathrm{CO}+\mathrm{Cl}+\mathrm{CO}$$

At the elevated temperatures of the equilibrium experiments, Cl­(CO) rapidly dissociates to yield the second Cl. The VUV probe light enters the reaction cell at a right angle to the photolysis laser. This probe light at 118.877 nm was generated via a YAG pumped (532 nm) dye laser (Quantel, Q-Smart – Sirah, Cobra-Stretch), giving output at 713.264 nm, which was doubled to produce between 4 and 8 mJ pulse^–1^ of 356.632 nm radiation. This UV light was focused (5 cm lens) into a tripling cell containing ca. 8 Torr of xenon. While the conversion efficiency to 118.877 nm is uncertain, it is estimated that there were between 10^10^ and 10^11^ photons pulse^–1^. The probe light passed through a MgF_2_ window on the reaction cell and intersected the photolysis beam in the center. The photolysis beam was typically 2 cm in diameter (a telescope shaped the output of the YAG laser), which maps out a larger volume than the expanding probe light. The photolysis laser was typically 80 mJ/pulse, and the volume defined by the intersection of the laser beams is ∼ 3 cm^3^. In a few experiments, the photolysis light was changed to the fifth harmonic, 213 nm, where its output energy was typically 10 mJ/pulse. The probe beam excited the Cl atom transition (4s ^2^D_3/2_ ← 3p ^2^P_3/2_), and the resulting fluorescence was monitored by a VUV photomultiplier (PerkinElmer, C 1311) mounted on top of the reactor, at right angles to the laser beams. This fluorescence from the Cl atom has a shorter lifetime than the pulse width of the laser beam, ∼6 ns. Therefore, this LIF signal cannot be time-resolved from the laser scatter. Fortunately, the LIF signal is significantly larger than the laser scatter for [Cl] > 10^10^ atom cm^–3^. This laser scattering is actually from the 357 nm UV light. Therefore, the detection limit for Cl atoms would be improved if the VUV light were separated from the UV light.

The probe light exited the reaction cell through a MgF_2_ window into a stainless-steel tube purged with N_2_. This VUV light passed along the tube until it hit a quartz window mounted at 45 deg. This directed the VUV light at right angles, where it passed through a VUV interference filter onto a second VUV photomultiplier. This second photomultiplier was used to determine the attenuation of VUV light from added C_2_H_4_. The signals from both photomultipliers were displayed on an oscilloscope (LeCroy 354), where it was observed that the pulse-to-pulse stability of the VUV light was ∼ 10%. The oscilloscope integrated these two signals, which were then passed to the PC for storage.

The photolysis and probe lasers were fired with a delay generator (BNC 555). For the majority of experiments, the laser repetition frequency was 10 Hz. In a few experiments, the repetition rate was set to 2 Hz to test the impact of product buildup; no significant change was observed. A custom LabVIEW program was used (via GPIB) to change the times between the lasers and transfer and store the oscilloscope signals. Typically, 220 time points were used to record a Cl atom kinetic trace, where 10% of the points were before time zero. After a scan, the process was repeated to provide the averaging. Typically, 5–15 scans were required before the trace was saved. As more C_2_H_4_ was added the VUV is attenuated via absorption, and more scans were required to maintain quality. The data were recorded such that the standard deviation of the signal could be recorded at each time point and used to weight the data fitting (see below).

A gas blackened bulb (5 L) of oxalyl chloride was prepared, ca. 1%. C_2_H_4_ (99.9%, Air Products), Ar (99.995, BOC), He (99.999, BOC), and N_2_(99.995, BOC) were used directly from the cylinder without further purification. At elevated temperatures, two pressures were used: ∼35 and ∼100 Torr, where the total flow was approximately the same, ∼100 cm^3^ s^–1^. This ensures the volume defined by the overlap of the laser beams (∼ 3 cm^3^) is swept before the next laser pulse, and the residence time in the reaction cell is always about the same. The [(ClCO)_2_] was typically between 10^13–14^ molecules cm^–3^, which leads to [Cl] between 2 and 20 × 10^11^ atom cm^–3^. This concentration was much smaller than the added ethylene, which was added up to ∼3 × 10^15^ molecules cm^–3^. Ceramic heaters surrounded the reaction cell and heated the cell over the range 298–822 K. The output of thermocouples mounted in the center of the cell was fed into temperature controllers, where the uncertainty in *T* was typically ± 1 K. However, it is noted that this is precision and not accuracy.

### 2.2 Ab Initio—Rate Theory

The potential energy surface for reaction R1 was evaluated by optimizing the reactants, products, transition states and potential intermediates using the post Hartree–Fock (HF) couple cluster with single and double excitation and perturbative triples approach (CCSD­(T))^22^ in combination with the ma-def2-TZVPP^23^ and aug-cc-pVDZ basis sets^24,25^ in ORCA 5.0.4.^26^ Harmonic vibrational frequencies (and gradients) were calculated numerically—scaled by 0.96—and the zero point energies were calculated from the combination of 3/8ths of the scaled harmonic and 5/8ths unscaled harmonic zero point energies as recommended by Csonka et al.^27^ Single point energy corrections were then calculated using CCSD­(T) extrapolated to CBS limit from aug-cc-pVDZ to QZ.^28–30^ Due to the potential impact of relativistic effects due to the presence of a third row element (Cl), second-order Douglass–Kroll–Hess relativistic corrections (DKH)^31^ were combined with all-electron CCSD­(T), along with the appropriate basis sets for DKH calculations aug-cc-pVXZ-DK (X = D to Q). Here, the DKH–CCSD­(T) energies were extrapolated to the CBS limit using a mixed Gaussian and exponential scheme.^32,33^ Two further sets of CCSD­(T) single-point energy corrections were calculated using the explicitly correlated CCSD­(T)-F12c with the cc-pVXZ-F12 basis sets extrapolated to the CBS limit between DZ and QZ. For the relativistic corrected DKH–CCSD­(T)-F12c calculations, appropriate basis sets were generated by decontraction of the cc-pVXZ-F12 basis sets. For the CCSD­(T)-F12c calculations in ORCA, along with the orbital basis set, a near-complete c-pVXZ-F12-CABS basis set was used (where X matched that of the orbital basis set). The implementation of CCSD­(T)-F12 used included a Resolution of the Identity (RI) approximation^34–37^ on the F12 component, which necessitated the inclusion of an additional auxiliary basis set here, aug-cc-pVnZ/c,^38^ where n was maintained 1 zeta level higher than the corresponding orbital basis set.

The DKH–CCSD­(T)/CBS//CCSD­(T)/ma-def2-TZVPP and DKH–CCSD­(T)/CBS//CCSD­(T)/aug-cc-pVDZ stationary points along the reaction surfaces were incorporated into MESMER version 7.1. In this rate theory analysis of the system using MESMER,^39^ the reaction was considered as two separate reactions: low-temperature adduct formation and high-temperature abstraction channel. In principle, both reactions can be solved simultaneously, but this massively increases the time to run the calculation. In a few simulation calculations, it was shown that the system can be separated into low and high-temperature regimes without any loss of accuracy. At temperatures below 500 K, adduct formation dominates the kinetics, and MESMER has been used to fit to the measured association and dissociation rate coefficients, [Disp-formula R1a] and 5, from this study and the literature. As this association occurs on a barrierless PES, the pragmatic method of inverse Laplace transformation (ILT) was used to calculate *k*_1a_ by adjusting the following parameters: A and *n* (*Ea* = 0, as there is no barrier) in a modified Arrhenius description of the high-pressure limit for the association. The other parameters that were adjusted by MESMER in the data fitting were the binding energy of the adduct, Cl–C_2_H_4_:ZPE, Δ*H*_R1a_^o^, which sensitively controls *k*_–1a_, and the energy transfer parameters, Δ*E*_down_, as this association reaction is in its pressure-dependent regime, where there are energy transfer parameters for each buffer gas. In this MESMER analysis, the low-frequency torsion in the adduct was treated as a 1-D hindered rotor, as this is a better description than a harmonic oscillator.

At temperatures greater than 500 K, the kinetics are dominated by abstraction.^16^ A transition state (TS) that was below the energy of the products was identified. In such circumstances, the rate coefficient is not wholly controlled by the energy barrier; entropy plays a significant role, and the position of the TS moves with temperature (variable). MESMER does not have the facility to vary a defined transition-state. The pragmatic approach to overcome this variational TS is to remove it from the MESMER reaction scheme and have an ILT reaction from the reactants to the complex—Complex-1—where the ILT barrier is fixed to the calculated barrier 23.4 kJ mol^–1^. The reaction from complex—Complex-1—to the products used Inverse ILT, where it was observed that these parameters had little effect on the abstraction rate coefficient, and so were fixed to close to the gas-kinetics frequency. In the discussion, the reverse abstraction reaction is considered, as there are experimental data,^17^ and it is another way to pinpoint the transition-state. While the ILT approach is a pragmatic way to allow for a variable transition-state via data fitting, it is not predictive and does not provide mechanistic information on the reaction. To provide insight into this abstraction reaction, variational transition state theory calculations were carried out using the software KiSThelP.^40^ The input for these calculations is provided by DKH–CCSD­(T)/CBS single-point energy corrections for many points along the intrinsic reaction coordinate (IRC) mapping the direct abstraction pathway, where at each point the vibrations are calculated. This input information allows the enthalpy and entropy to be calculated at each point, which is used to identify the minimum in the free energy of activation, Δ*G* ^‡^, the transition state for a given temperature.

## 3 Results

### 3.1 Room Temperature Kinetics on R1

All experiments on R1 were carried out under pseudo-first-order conditions with [C_2_H_4_] ≫[Cl]_0_. At room temperature, there is no decomposition of the addition complex, and the rate of removal of Cl is given by

E1 $$\frac{-d\left[\mathrm{Cl}\right]}{dt}=k_{1}\left[C_{2}H_{4}\right]\left[\mathrm{Cl}\right]+k_{\mathrm{loss}}\left[\mathrm{Cl}\right]$$

Given that [C_2_H_4_]≫[Cl]_0_ and that the LIF signal (*I*_f,Cl_) is proportional to the Cl atom concentration, 3 can be integrated to give:

E2 $$I_{f,\mathrm{Cl}}\left(t\right)=I_{f,\mathrm{Cl}}\left(0\right)e^{-k_{1}^{\prime }t}$$

where *I*_*f,Cl*_(*t*) is the signal at time *t* after the laser fires, *I*_*f,Cl*_(0) is the initial LIF signal, and *k*_1_′ = *k*_1*a*([M])_[C_2_H_4_] + *k*_loss_, where *k*_loss_ is the rate coefficient for all other first- or pseudo-first-order loss processes (e.g., diffusion or reaction with any impurities).

The inset of Figure 1 shows a typical room temperature decay and fit to eq 4. *k*_1a([*M*])_ was obtained by repeating the experiment at varying [C_2_H_4_], and *k*_1a([*M*])_ is the gradient of a plot of *k*_1_′ vs [C_2_H_4_].

**Figure 1:**
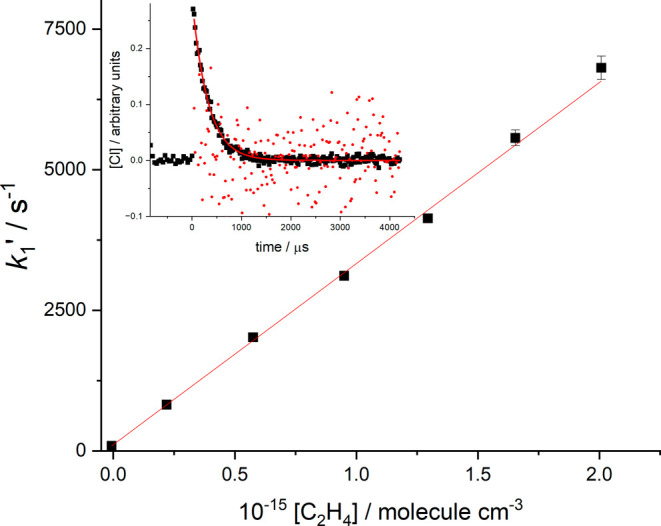
Bimolecular plot for Cl + C_2_H_4_ at room temperature (RT) with N_2_ as the buffer gas, where the total pressure was 9.4 Torr. The slope, *k*_1a([*M*])_, is equal to (3.29 ± 0.06) × 10^–12^ cm^3^ molecule^–1^ s^–1^, where the error is 2σ. The inset is the Cl time trace that corresponds to the point [C_2_H_4_] = 9.50 × 10^14^ molecule cm^–3^ from the bimolecular plot, and the circles are the residuals (x10) of the fit (red) to the data. The photolysis laser for R*T* experiments used the 5th harmonic at 213 nm.

As reaction 1a is pressure dependent, experiments were repeated at different pressures, as shown in Table 1. In addition, a few room temperature experiments used 213 nm to photolyze oxalyl chloride, 2. This was to check whether ClCO from [Disp-formula eq2] had a significant impact on the kinetics. ClCO is known to form in significant amounts at ≥ 248 nm, and its kinetics^41^ can potentially overlap R1. Figure 2 shows the room temperature values and the good agreement with the literature,^11–13,42,43^ for similar bath gases (Wallington et al. performed some experiments in air and N_2_, obtaining the same results within errors), demonstrating the validity of the Cl atom detection method. Our values with argon as the bath gas are slightly lower, but this is consistent with less efficient energy transfer of argon compared to nitrogen. A full analysis of the temperature and pressure dependence is presented below.

**Figure 2:**
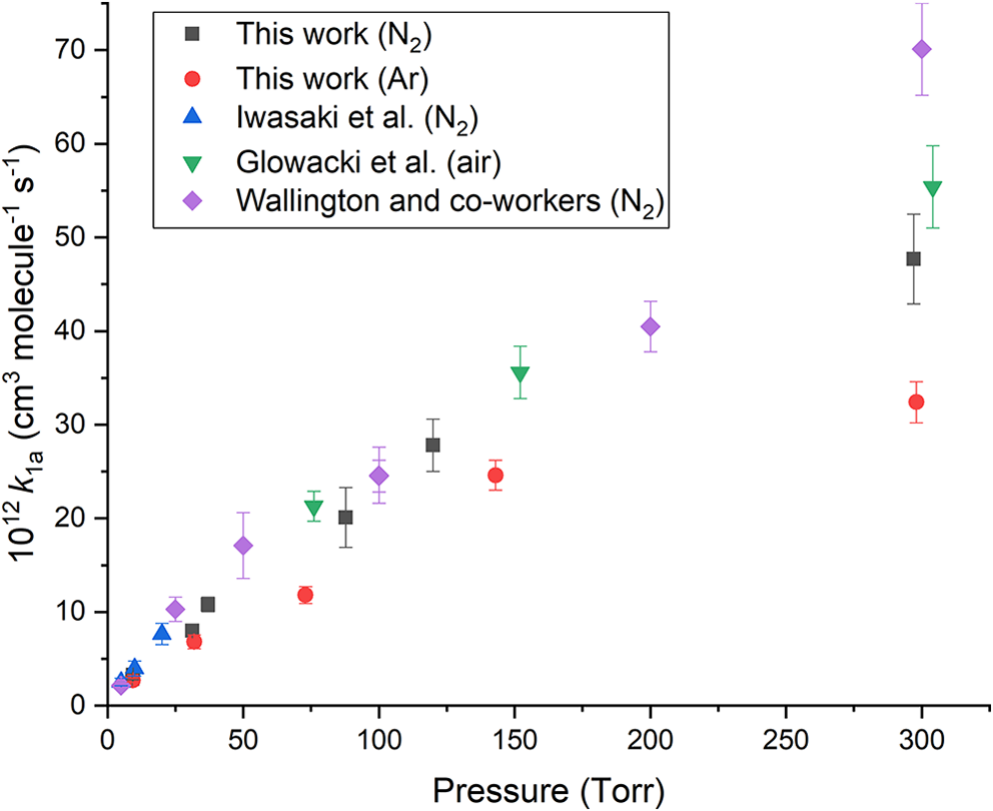
Comparison of rate coefficients for the reaction Cl + C_2_H_4_ → ClCH_2_CH_2_. Black squares, this work with M = N_2_; red circle, this work with M = Ar; blue triangle, Iwasaki et al.^6^ M = N_2_; green inverted triangles; and Glowacki et al.;^13^ purple diamonds. Wallington and co-workers^11,12^ M = air.

**Table 1:** Rate Coefficients for Reaction R1 at Low and High Temperatures^a^

*T*/K	pressure/torr	10^12^ *k*_1a([*M*])_/cm^3^ molecule^–1^ s^–1^	10^13^ *k*_1b_/cm^3^ molecule^–1^ s^–1^
296	31.9	6.82 ± 0.72	
296	73	11.8 ± 0.90	
296	143	24.6 ± 1.6	
296	298	32.4 ± 2.2	
296	9.4^b^	2.70 ± 0.18	
296	9.2 (N_2_)	2.86 ± 0.08	
296	31.2 (N_2_)	7.98 ± 0.38	
296	87.8 (N_2_)	20.1 ± 3.2	
296	297 (N_2_)	47.7 ± 4.8	
296	9.4 (N_2_)^b^	3.30 ± 0.12	
296	37.0 (N_2_)^b^	10.8 ± 0.78	
296	119.9 (N_2_)^b^	27.8 ± 2.8	
523	36.2		1.2 ± 0.2
623	36.4		3.4 ± 0.2
723	36.3		7.1 ± 2.3
822	36.3		6.6 ± 0.6

a At these temperatures, R1 exhibited single-exponential Cl removal kinetics, and rate coefficients were determined via bimolecular plots. The buffer gas was argon, except where indicated. Quoted errors are equal to 2σ.

b Photolysis used 213 nm.

### 3.2 High-Temperature Kinetics on R1

At temperatures >500 K, the Cl removal exhibited single-exponential behavior, and *k*_1_′ can be obtained via E2. At these temperatures, *k*_1_′ is equal to = *k*_1*b*_[*C*_2_H_4_] + *k*_*loss*_, and a bimolecular plot yields *k*_1b_. These abstraction kinetic results are also reported in Table 1.

### 3.3 Reactivity Measurements

Having demonstrated that the LIF scheme returns reliable Cl kinetics, we can now take advantage of the fact that there is minimal absorption of probe radiation by oxygen, allowing us to make the first direct Cl reactivity measurements. Ambient air was sampled from outside the Chemistry Laboratory through 30 m of 0.25 cm i.d. Teflon tube at a flow rate of 699 standard cubic centimeter per minute (SCCM) into the reaction cell. Also, flowed into the reaction cell was N_2_ at ∼ 2500 SCCM and ∼ 1 SCCM of the ∼ 1% oxalyl chloride, the Cl atom precursor. The reaction cell was held at 451 Torr to account for contributions from pressure-dependent reactions; the mass flow controller that sampled the air would not flow at a higher total pressure. This arrangement led to 0.22 of the gas in the cell being from sampled air. Reactivity measurements were taken in the same way as described above for the kinetic measurements. Figure 3 shows an example of the data obtained, where the Cl removal rate coefficient, *k*_removal_, is 145 s^–1^. To account for the removal in the absence of the air sample, the sample flow was turned off and the N_2_ flow turned up; therefore, the total flow and pressure remained the same. *k*_removal_ for N_2_ was 48 s^–1^. The difference between the sampled air and N_2_ is 97 s^–1^. Scaling this result to atmospheric pressure leads to Cl reactivity, *k*_reactivity_, equal to 725 s^–1^, where the main error is the reproducibility in determining *k*_removal,N2_. Over a 3 h of air sampling, *k*_reactivity_ varied no more than 20%. It was noted that N_2_ quenching of the LIF signal was not significant, and O_2_ absorption was not a major issue when carrying out these experiments. The main problem is VUV absorption by water vapor, H_2_O. The Mainz absorption database^44^ has the VUV H_2_O cross-section at 119 nm equal to ∼ 10^–17^ cm^–2^. This prevents sampling high-humidity air at 1 atm but does not prevent the determination of *k*_reactivity_ using the procedure outlined above. A shorter path length reaction cell or selective removal of H_2_O from the air sample may allow *k*_reactivity_ measurements at atmospheric pressure.

**Figure 3:**
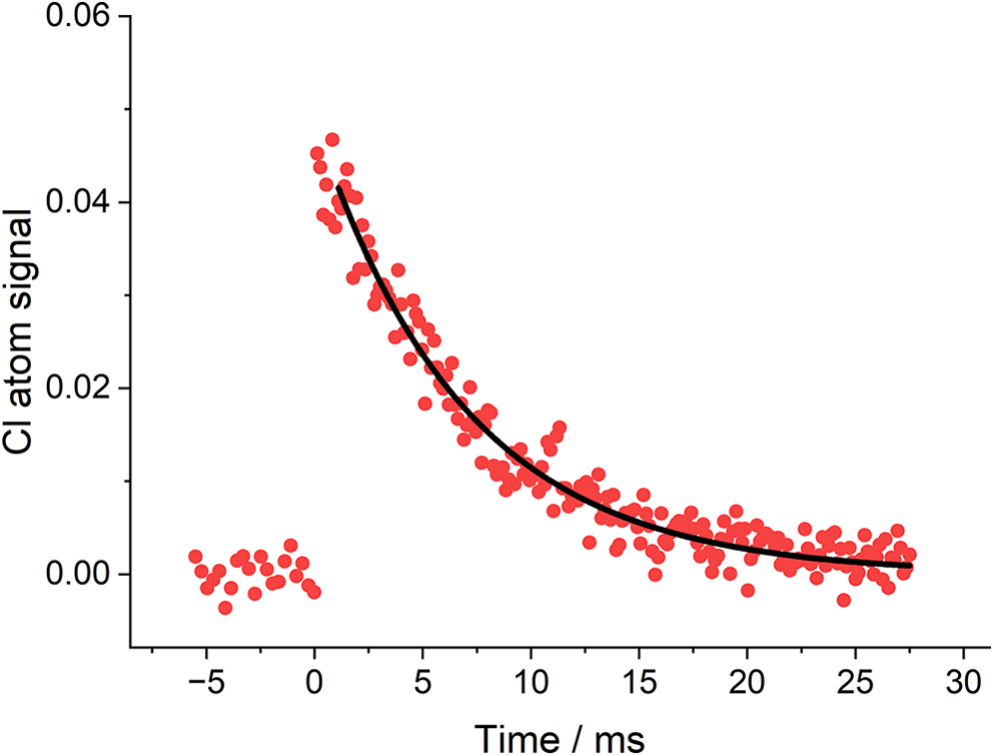
Typical Cl reactivity measurement recorded with air sampled from outside the Chemistry Laboratory. Measurements were taken at a total pressure of 451 Torr. The decay is (144.8 ± 3.6) s^–1^ corresponding to a reactivity value of 725 s^–1^ (*k*_reactivity_) at atmospheric pressure, see text for details.

This observed Cl reactivity is significantly higher than has been observed for OH reactivity in urban environments (e.g., an average of 30–40 s^–1^ in central Beijing^45^); however, Cl atoms typically react an order of magnitude faster than OH with most VOC, and sampling directly outside a chemistry laboratory is not typical of the urban background. Further work is planned to develop a field instrument.

### 3.4 Observation of Equilibrium Behavior for R1

At temperatures above 390 K, the kinetic traces were nonsingle exponential, and an example is shown in Figure 4. This behavior is consistent with the onset of equilibrium behavior that has previously been observed at Leeds in the reactions of H and OH with ethylene.^46,47^ The main difference with the present system is that equilibrium is observed at a much lower temperature. However, this is consistent with theory that predicts a much smaller binding energy for Cl–C_2_H_4_ ^18^ compared to HO–C_2_H_4_ ^46^ and H–C_2_H_4_ ^47^

**Figure 4:**
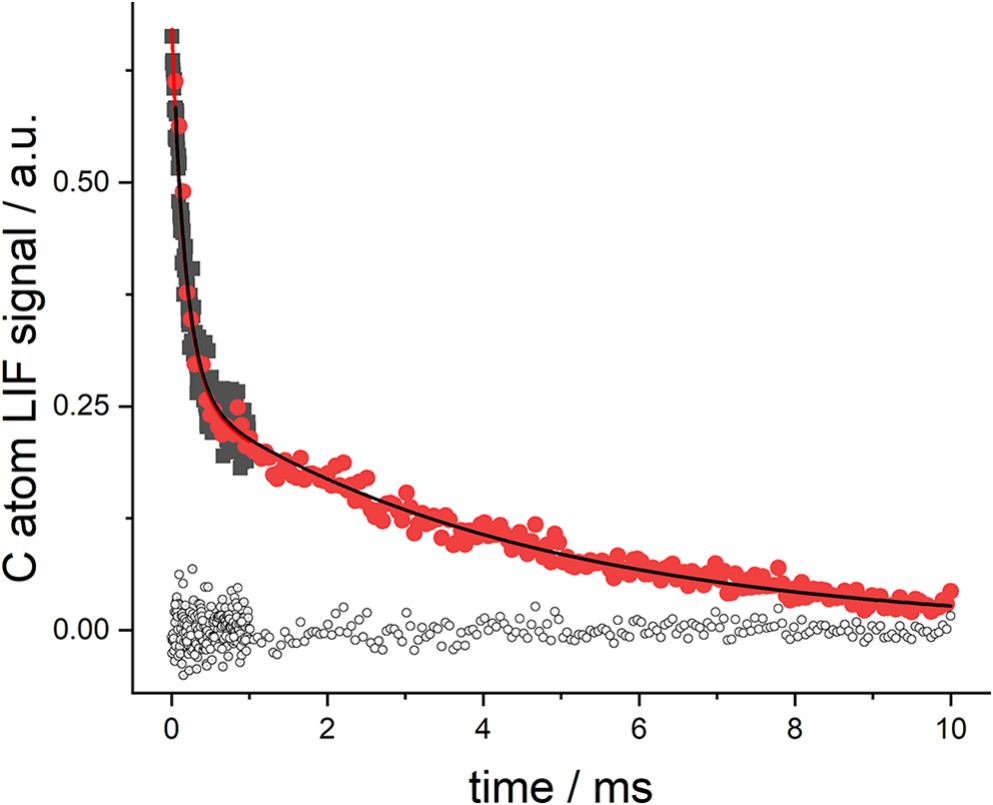
An example of a kinetics trace exhibiting equilibrium behavior in the reaction between Cl and C_2_H_4_ at a temperature of 430 K. The total pressure (Ar) was 99.3 Torr, and [C_2_H_4_] was equal to 7.06 × 10^14^molecules cm^–3^. For a given ethylene concentration, two time-traces were recorded to better define the multiple time constants of the system, red and black traces. The solid lines are the fits to the data, and the circles at the bottom are the residuals. In the data analysis, the rate coefficients were shared, but the initial [Cl]_0_ was local to each trace.

The analysis of these traces used the equilibrium scheme:

$$\mathrm{Cl}+C_{2}H_{4}\overset{k_{1a\left(M\right)}\;}{\to }\mathrm{Cl}-C_{2}H_{4}$$ R1a

R1-a $$\mathrm{Cl}-C_{2}H_{4}\overset{k_{-1a\left(M\right)}\;}{\to }\mathrm{Cl}+C_{2}H_{4}$$

In the absence of added ethylene, the chlorine atoms are slowly removed from the system, this is mainly via diffusion—less than 50 s^–1^—as there is no evidence that Cl reacts with the precursor, oxalyl chloride:^48^

Rloss $$\mathrm{Cl}\overset{k_{\mathrm{loss}}\;}{\to }\mathrm{loss}$$

However, analysis indicates that there is an additional loss of Cl–C_2_H_4_ from the system. The wavelength of the probe laser was tuned to check for H atom formation^21^ but none were observed; this is consistent with our *ab initio* calculations, see below. This additional removal rate was typically a few 100s s^–1^ and was generally independent of temperature. Based on known small leaks into the system, this behavior is not inconsistent with Cl–C_2_H_4_ reacting with O_2_. This behavior was also observed in our OH + C_2_H_4_ study.^46^ This additional reaction was modeled via:

R2 $$\mathrm{Cl}-C_{2}H_{4}\overset{k_{2}\;}{\to }\mathrm{loss}$$

At temperatures >500 K, it is known that the abstraction to vinyl becomes dominant:^16^

R1b $$\mathrm{Cl}+C_{2}H_{4}\overset{k_{1b}\;}{\to }\mathrm{HCl}+C_{2}H_{3}$$

This reaction scheme, R_1a_, R_–1a_, R_loss_, R_2_, and R_1b_, was formulated into ordinary differential equations and solved for the appropriate initial conditions. Figure 4 shows an analysis of a pair of traces for a given ethylene concentration. To improve the reliability of the parameters in the data analysis, which is more of a problem for multiexponential traces, global analysis was performed for a given temperature and pressure. Typically, 6–8 pairs of data were used in this global analysis, where for each pair the ethylene concentration was assigned to define *k*_1a_′ (=*k*_1a(*M*)_ × [C_2_H_4_]) and *k*_1b_′ (=*k*_1b_ × [C_2_H_4_]). All of the rate coefficients in this analysis were global, where *k*_1b_ was allowed to float but constrained so that it did not exceed the literature values measured by Pilgrim and Taatjes^16^ by a factor of 2. Also, fixing *k*_1b_ to the values given by Table 5 yielded essentially the same parameters. At these equilibrium temperatures, *k*_1b_ typically contributed less than 10% to R1. Each trace had its own chlorine atom concentration, [Cl]_0_. This analysis was carried out using the software ORIGIN, and the results are given in Table 2.

**Table 2:** Forward (*k*_1a([*M*])_) and Reverse (*k*_–1([*M*])_) Rate Coefficients Determined from the Global Analysis of the Cl + C_2_H_4_ Time Traces^a^

*T*/K	pressure/torr	10^12^ *k*_1a([*M*])_/cm^3^ molecule^–1^ s^–1^	*k*_–1a([*M*])_/s^–1^
393	36.4	3.13 ± 0.05	157 ± 11
417	36.2	2.42 ± 0.06	565 ± 26
441	36.4	1.95 ± 0.10	1775 ± 92
465	36.2	1.48 ± 0.09	3749 ± 251
490	36.3	0.78 ± 0.18	8967 ± 1586
490	36.3	0.82 ± 0.30	11696 ± 3652
409	98.8	7.09 ± 0.13	761 ± 24
430	99.3	5.21 ± 0.14	2119 ± 62
452	99.5	3.72 ± 0.24	5292 ± 262
472	99.3	3.37 ± 0.50	10848 ± 1164
423	49.7 (He)	2.57 ± 0.08	760 ± 46
473	49.8(He)	2.07 ± 0.40	5600 ± 816
430	100.2 (N_2_)	6.28 ± 0.22	2186 ± 84

a Additional loss of the adduct, *k*_2_, was floated and generally returned values of a few 100 s^–1^. *k*_1b_ was either allowed to float but constrained—see main text—or fixed to the values given in Table 5 without significantly changing the rate coefficients of this table. The fits to the data were excellent. The main buffer gas was argon, and this is indicated for other buffer gases. Quoted errors are equal to 2σ from the analysis. Estimates of schematic errors suggest that the accuracy of the rate coefficient is ∼ 10% based on the MESMER analysis.

### 3.5 Ab Initio Calculations

Four approaches to calculate the CCSD­(T)/CBS single-point energy were used, including the second-order Douglass–Kroll–Hess (DKH) relativistic correction to account for the relativistic errors for third-row atoms that become important for second-row atoms, such as Chlorine. The results are summarized in Table 3, where it can be seen that the energy of Cl–C_2_H_4_, the binding energy, varies over the range 76.9 to 79.8 kJ mol^–1^. Also observed from Table 3 is that including relativistic effects (methods 2 and 4) lowers the energy by ca. 1 kJ mol^–1^; this is pushing the binding energies closer to the experimental value, see below. The calculations carried out in this study returned a narrow range of energies for all the species considered in reaction R1, which implies these calculations are reasonably converged.

**Table 3:** Zero-Point Energy-corrected Relative Energies Calculated at the CCSD­(T)/CBS//CCSD­(T)/aug-cc-pVDZ and CCSD­(T)/CBS//CCSD­(T)/ma-def2-TZVPP Level^a^

	X//CCSD(T)/ma-def2-TZVPP (kJ mol^–1^)				X//CCSD(T)/aug-cc-pVDZ (kJ mol^–1^)				
structure	1	2	3	4	1	2	3	4	ATcT(σ)
Cl + C_2_H_4_	0.00	0.00	0.00	0.00	0.00	0.00	0.00	0.00	0.00
Cl–C_2_H_4_	–77.69	–76.85	–78.61	–77.86	–78.76	–77.95	–79.80	–78.94	
H elim TS-2	87.46	88.36	86.66	88.05^b^	87.17	88.06	86.30	87.82^b^	
H + C_2_H_3_Cl	62.12	63.05	62.04	62.70	62.36	63.28	62.21	62.44	64.91(0.29)
H transfer TS-4	92.19	93.20	91.30	93.28	91.12	92.13	90.16	91.18	
CH_3_CHCl	–94.29	–93.18	–95.61	–94.58	–95.18	–94.09	–96.56	–95.43	
H elim TS-3	74.20	75.13	73.42	74.77^b^	74.13	75.05	73.29	74.78^b^	
CH_3_+HCCl(S)	286.01	286.29	285.28	285.06	286.15	286.42	285.41	285.42	289.71(0.90)
CH_3_+HCCl(T)	312.83	313.72	311.44	312.28	312.73	313.61	311.32	312.27	315.59(0.90)
H Abs TS-1	22.72	23.37	23.01	24.06^b^	22.84	23.48	23.02	24.13^b^	
Complex-1	19.58	20.51	19.21	20.04	19.82	20.75	19.39	20.34	
HCl + C_2_H_3_	28.48	29.41	28.56	29.03	28.24	29.17	28.22	28.82	0.29

a Here, the CCSD­(T)/CBS single point energies were determined with 4 approaches to the couple cluster calculations (1) CCSD­(T), (2) DKH–CCSD­(T), (3) CCSD­(T)-F12c, and (4) DKH–CCSD­(T)-F12c.

b For species where DKH–CCSD­(T)-F12c/cc-pVQZ-F12 generated erroneous results DKH–CCSD­(T)-F12c/cc-pVTZ-F12 values are provided.

The overall potential energy surface that includes all of the species in Table 3 is given in Figure 5. Also included in Table 3 are values from the Active Thermodynamic Table (ATcT).^49^ While ATcT values are not available for all species, it is observed that these calculations are with 2–3 kJ mol^–1^; very reassuring. For the direct abstraction channel to HCl + C_2_H_3_, the calculated endothermicity is within 1 kJ mol^–1^ of ATcT.

**Figure 5:**
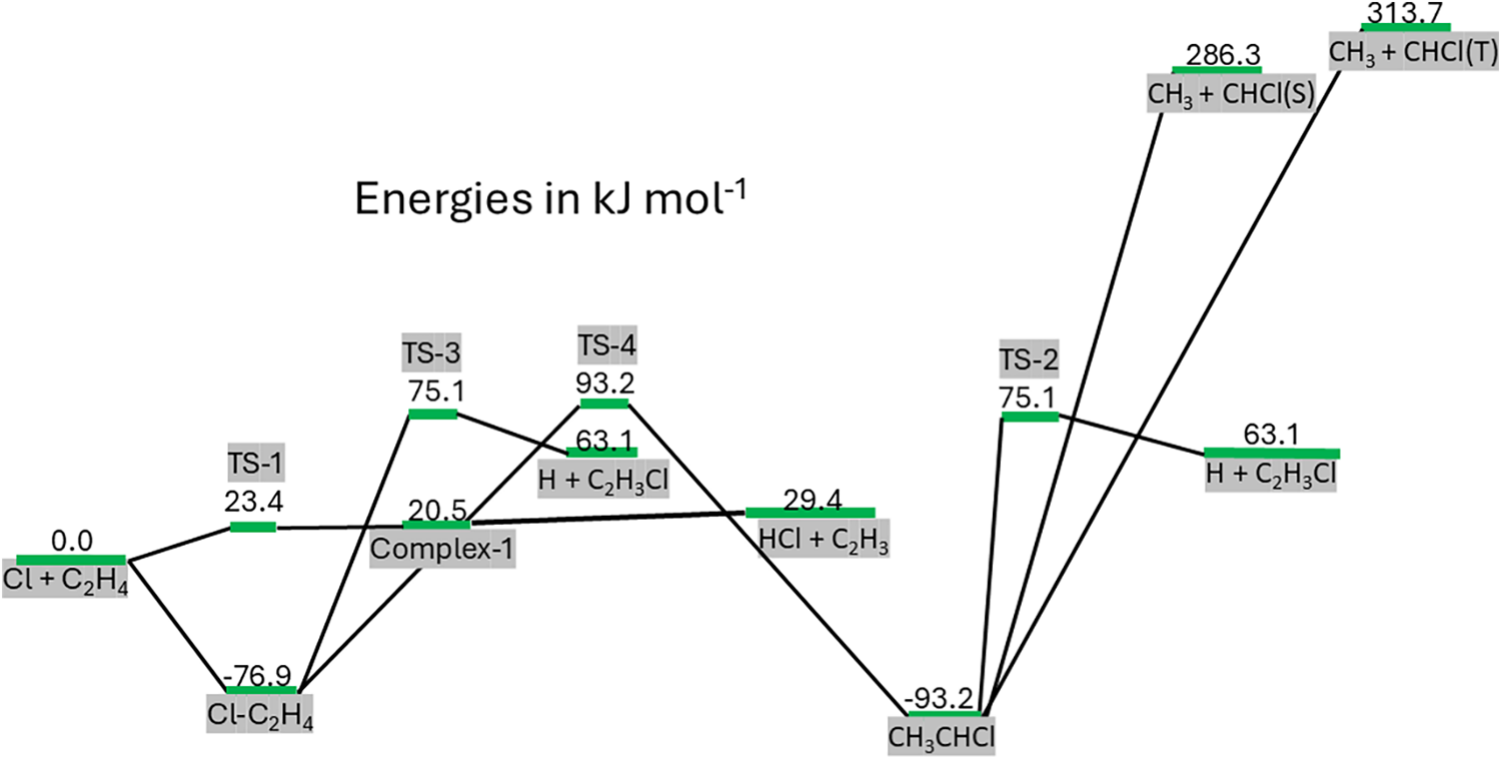
Potential energy surface (PES) explored on reaction R1, where the energies of each species are given in Table 3. Not all species contribute to the kinetics of R1, and consequently, a reduced subset of this PES is used in the analysis of the experimental data.

## 4 Discussion

### 4.1 Cl–C_2_H_4_ Binding Energy, ΔH_R1a_^o^

From the parameters in Table 2, it is readily observed that the redissociation rate coefficient, *k*_–1(*M*)_, increases with temperature, as expected, and similarly *k*_1a(*M*)_ decreases, as expected for a pressure-dependent reaction proceeding on a barrierless surface. The forward and reverse rate coefficients describe the equilibrium constant:

E​Q-R1a $$K_{c,1a}=\frac{k_{1a_{\left(M\right)}}}{k_{-1a_{\left(M\right)}}}$$

Dividing *K*_c,1a_ by *RT*, where *R* is the gas constant, converts it to *K*_p_, the equilibrium constant, in terms of partial pressures. A van’t Hoff plot of ln *K*_p_ versus 1/*T* is shown in Figure 6, where the best straight-line through the data yields the enthalpy (Δ*H*_R1a_^o^/*R*) and entropy (Δ*S*_R1a_/*R*) of the reaction at the average temperature of the equilibrium experiments, 429 K. While this is the best line through the data, it is well-known that it is more accurate to calculate the entropy from the properties for the reacting species and then perform the van’t Hoff plot with this fixed Δ*S*_R1a_; this is called the third law method. These two van’t Hoff plots are shown in Figure 6, where the third Law assigned Δ*H*_R1a_^o^ = 75.8 ± 0.3 kJ mol^–1^.

**Figure 6:**
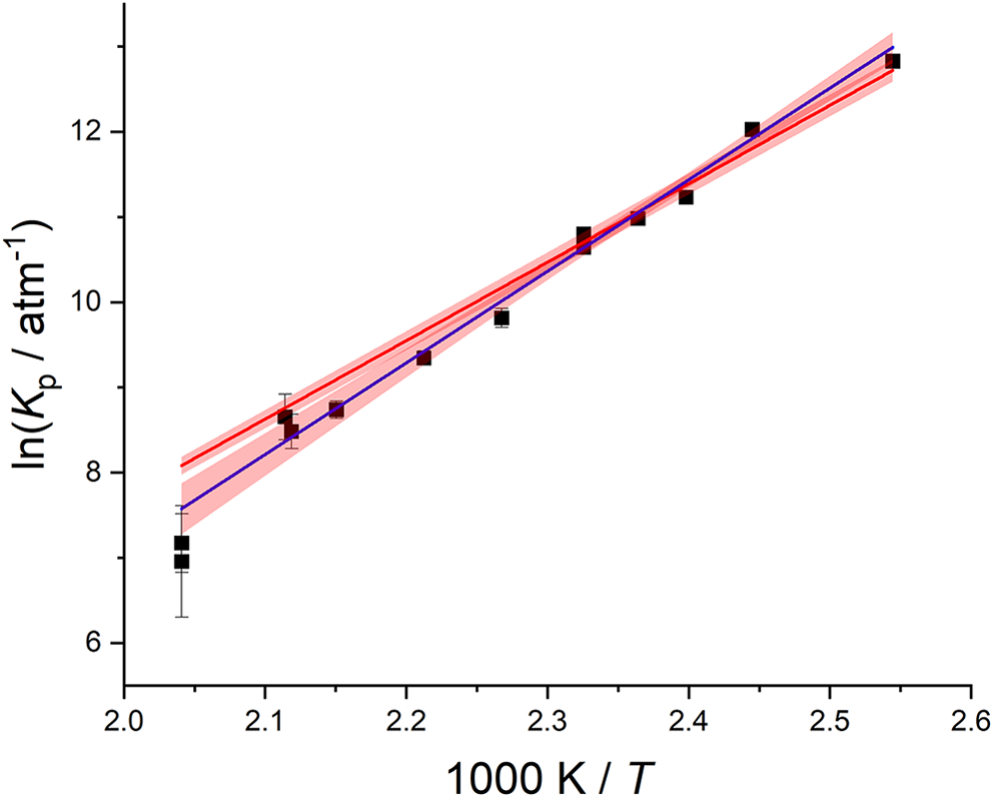
van’t Hoff plot of the equilibrium data determined in this study. There are two straight lines: the 2nd Law, where entropy (−119.3 ± 7.9 J mol^–1^ k^–1^) and enthalpy (89.3 ± 3.3 kJ mol^–1^) are adjusted, blue, and the 3rd Law, where the entropy is fixed to the model value (−89 J mol^–1^ k^–1^) and this yields an enthalpy equal to 76.5 ± 0.2 kJ mol^–1^, red.

To understand the system in more detail, the Master Equation Solver MESMER^39^ was used to fit to all of the data given in Tables 1 and 2 (Leeds) and also including the room temperature literature data, using the molecular properties description of the species involved in the association step of R1, see Figure 7. This data fitting analysis assigns the enthalpy of reaction, but it is also able to assign the energy transfer parameters of the gases used in this study: argon, helium, and nitrogen. These results are summarized in Table 4. In Table 4, the value Δ*H*_R1a_^o^ at the temperature of the equilibrium experiments is in excellent agreement with the third Law analysis shown in Figure 6. The errors reported in Table 4 are from using the rate coefficients of Tables 1 and 2, where errors are assigned as 10%, unless the stated errors in Tables 1 and 2 are greater. Using these 10% errors results in the χ^2^/ pts from the MESMER analysis of ca. 1, which implies the errors quoted in Table 4 are 1σ. The errors quoted in Tables 1 and 2 are 2σ from the fit to the trace data, but this does not consider systematic errors, which are estimated to be ca. 10%. As expected, from Table 4, it can be seen that including the literature data has a minimal impact on the Δ*H*_R1a_^o^.

**Figure 7:**
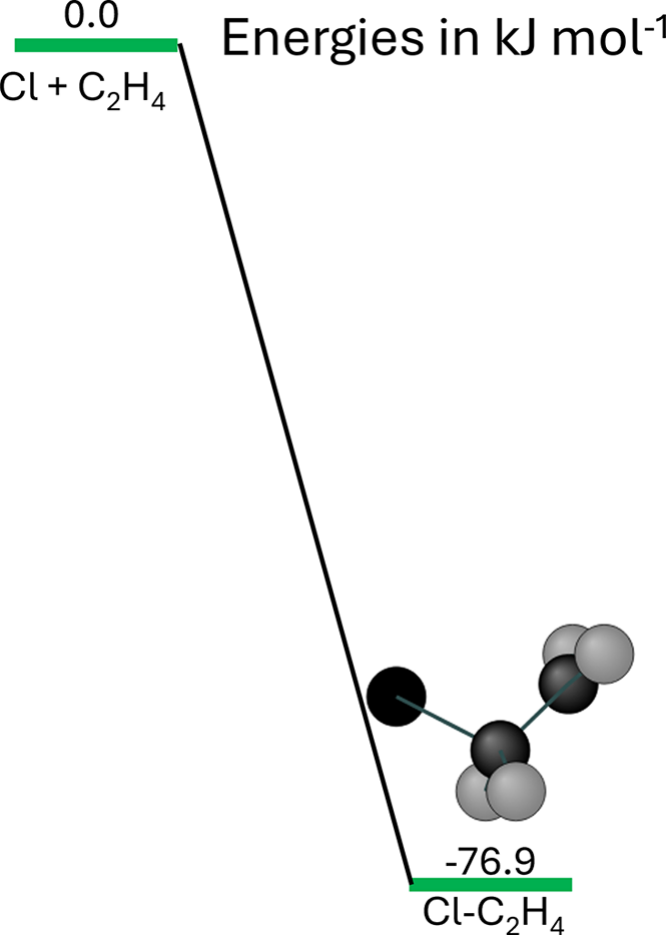
Association PES for R1 ([Disp-formula R1a]) and used in the MESMER analysis to determine the binding energy of Cl–C_2_H_4_, where a 1-D hindered rotation was used to describe its lowest vibration.

**Table 4:** MESMER Results from Fitting the Rate Coefficients in Table 1, Using 10% Errors^a^

	leeds data	leeds and literature	units
Δ_r_ *H*_0,R1a_^o^	–74.0 ± 0.16	–74.1 ± 0.30	kJ mol^–1^
Δ_r_ *H*_298,R1a_^o^	–76.8 ± 0.2	–76.9 ± 0.30	kJ mol^–1^
Δ_r_ *H*_429,R1a_^o^	–76.4 ± 0.2	–76.5 ± 0.2	kJ mol^–1^
A: *k*_*1a*_^*∞*^ = A × (*T*/298)^−0.2^	2.0 ± 1.1 × 10^–10^	3.27 ± 0.46 × 10^–10^	cm^3^ molecule s^–1^
Δ*E*_down,298_(Ar)^b^	187 ± 25	169 ± 14	cm^–1^
*n*	0.68 ± 0.28	0.82 ± 0.23	
Δ*E*_down,298_(N_2_)^c^	224 ± 26	236 ± 12	cm^–1^
Δ*E*_down,298_(He)^c^	116 ± 10	113 ± 8	cm^–1^
χ^2^/degrees of freedom	1.40	1.50	

a Errors are equal to 1σ. Literature includes data from Atkinson,^42^ Kaiser,^12^ Wallington,^11^ Coquet,^50^ Stutz,^14^ Iwasaki,^6^ and Glowacki^13^ (minus the low-pressure points).

b Δ*E*_down_,= Δ*E*_down,298_ × (*T*/298)^*n*^ for Ar, there are sufficient points to define both Δ*E*_down,298_ and *n*.

c Δ*E*_down_,= Δ*E*_down,298_ × (*T*/298)^*n*^. For N_2_, *n* is fixed as 0.25, and for He, *n* is fixed as 1.0.

Braña et al.^18^ have previously calculated the binding energy of the adduct of Cl–C_2_H_4_ and reported a range of values from 70 to 81 kJ mol^–1^, where no calculation was favored. It is estimated that these calculations are accurate to no better than 10–15 kJ mol^–1^. The level of calculations in this study represents a significant improvement compared to Braña et al.^18^ and the results are summarized in Table 3. From this table, a narrow range of binding energies is calculated for Cl–C_2_H_4_, 76.9 to 79.8 kJ mol^–1^. From Table 4, the MESMER assigned value from the experimental data from this study is 74 kJ mol^–1^, and a similar value of 75 kJ mol-1 was determined from the experiments by Knyazev et al.^17^ Therefore, the present calculations appear to have improved the accuracy of the Cl–C_2_H_4_ binding energy to within 4 kJ mol^–1^. Also from Table 3, it is noted that the Douglass-Kroll-Hess (DKH) relativistic correction calculations, 2 and 4, are ca. 1 kJ mol^–1^ closer to the experimental binding energy.

### 4.2 *k*_1a(*M*)_: Association Kinetics

The removal kinetics of Cl atoms with ethylene, R1, have been determined previously^6,11–14,42,50^ mainly in experiments close to room temperature. This additional literature *k*_1a(*M*)_ data have been added to the MESMER analysis (Leeds and literature), and the χ^2^/pt is close to the Leeds data alone, with no obvious systematic behavior, see Table 4. These extra data, especially Wallington et al.^11^ that explored the reaction to high pressures, better pinpoint the high-pressure limit, *k*_1a_^∞^. The value of *k*_1a_^∞^ given in Table 4 is in excellent agreement with Kaiser et al.^12^ For the relative rate study by Glowacki et al.^13^ the data fitting revealed that the rate coefficients below bath gas density of 5 × 10^18^ molecules cm^–3^ were systematically high, increasing to about double the predicted rate coefficient at their lowest pressure, 3.6 × 10^17^ molecules cm^–3^. A possible explanation for this is that the reagents had varying contributions from wall losses, which shows up significantly at low pressures, where the small rate coefficient means a longer experimental run. All data from Glowacki et al.^13^ below 5 × 10^18^ were excluded from the analysis. Overall, this literature data compliments the data from this study and returns consistent energy transfer parameters, where He < Ar < N_2_. *k*_1a[*M*]_ has little impact on the binding energy; therefore, this parameter is returned almost unchanged. The MESMER file for this analysis is given in the SI.

### 4.3 *k*_–1a(*M*)_: Redissociation Kinetics

The redissociation rate coefficient, *k*_–1a([*M*])_, has only been measured previously by Knyazev et al.^17^ These experiments were not Cl-initiated but involved the photolysis (248 nm) of a suitable precursor (CH_2_ClCH_2_I) to directly generate Cl–C_2_H_4_. These *k*_–1a([*M*])_ values were determined between 440 and 460 K and were added to the MESMER analysis. These additional values were reasonably fitted, but on average, these values were 20–30% systematically higher than the fit. A possible explanation for this systematic deviation is that the measured Cl–C_2_H_4_ loss also contained wall losses, which were between 20–50% of the assigned *k*_–1a([*M*])_ values. In addition, these wall losses were determined at 350 K, at temperatures where *k*_–1a([*M*])_ was small. This systematic deviation has only a small effect on the determination of the enthalpy of reaction R1. Knyazev et al. reported a value of 74.8 kJ mol^–1^ for the enthalpy of reaction for R1 at 0 K, which is in excellent agreement with the present study, see Table 4.

### 4.4 *k*_1b_: Abstraction Kinetics

This study has shown that below 500 K, addition will obscure the direct abstraction reaction channel:

R1b $$\mathrm{Cl}+C_{2}H_{4}\overset{k_{1b}\;}{\to }\mathrm{HCl}+C_{2}H_{3}$$

This was previously noted in the study by Pilgrim and Taatjes^16^ where the formation of HCl was observed to be nonexponential at temperatures below 500 K and consistent with secondary formation via reactions such as Cl + ClCH_2_H_2_ → HCl + C_2_H_3_Cl which they assumed would have similar kinetics to Cl + C_2_H_5_.^51^ For this reason, all reported HCl measurements at lower temperatures are not included in our analysis. In Table 1, the four *k*_1_ values above 500 K represent abstraction, as the temperature is too high for the adduct to have a significant lifetime. These rate coefficients are considerably smaller than the removal rate coefficients measured at lower temperatures, and are consistent with the values reported by Pilgrim and Taatjes.^16^ It is noted that Pilgrim and Taatjes assigned their rate coefficients, *k*_1b_, by multiplying it by the HCl yield, where the yield at 550 K is ca. 0.5 and increases to ca. 1.0 at 800 K. The reason for this increasing HCl yield with temperature is unclear. In our *ab initio* calculations, the full PES, see Figure 5, has been explored via simulations and loss from Cl–C_2_H_4_ via isomerization or H atom elimination was observed to be minimal over all temperature and the HCl yield was 1.0 at all *T* > 500 K. Therefore, there might be up to a factor of 2 uncertainty in these rate coefficients, which is more than the difference between our measurements (total removal) and Pilgrim and Taatjes^16^ (HCl formation).

Reaction R1abs is endothermic and is best estimated to be 28.75 kJ mol^–1^ according to the Active Thermodynamic Tables, ATcT.^49^ Our calculations are in excellent agreement, within 1 kJ mol^–1^ for all the calculations given in Table 3. R1 kinetics above 500 K are essentially abstraction, and the PES given in Figure 5 can be approximated to that given in Figure 8. From Figure 8, the transition state (TS-1) is below the energy of the products. The position of the transition-state is significantly controlled by entropy and hence changes with temperature. Under these circumstances variational transition-state theory (v-TST) should be used.

**Figure 8:**
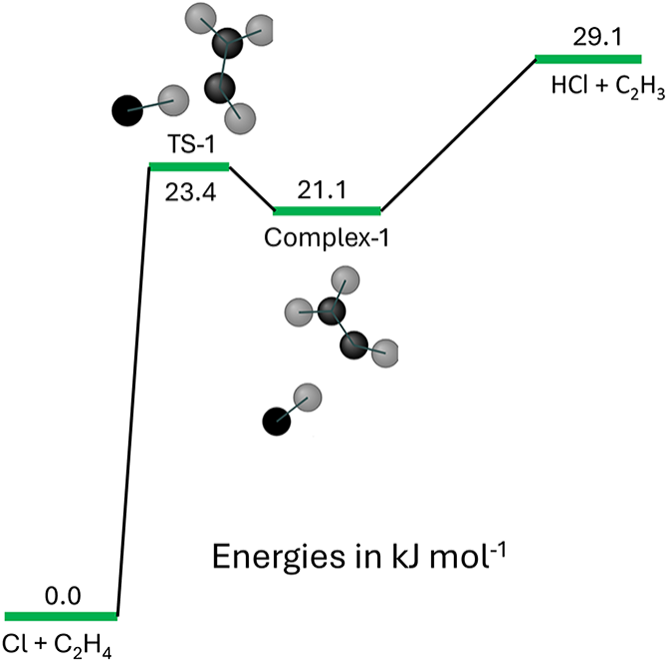
Reduced abstraction PES surface that dominates the overall reaction >500 K. The transition-state (TS-1) is below the energy of the products. This leads to entropy significantly contributing to the position of the transition-state along the PES, as a function of temperature. This means that variational TS is required to describe the system. ILT can be used to capture this variational TS by fitting to the experimental data. Alternatively, variational TST was used to directly explore the rate coefficient using software KiSThelP.

Such reaction profiles are not that common for reactions with a significant endothermicity; however, the X + RH = HX + R family of reactions (where X = Cl, Br, I) have this reaction profile. It has been more common to study the system from the R + HX direction (i.e., C_2_H_3_ + HCl for R1b) as the smaller, negative activation energy means that the reaction can be studied using a single experimental technique over a wide range of temperature.^52,53^ The observations of negative activation energies, and the associated impact on radical heats of formation, were a controversial issue,^54–59^ and this is still an active area of research.^60^ In this section, we combine our measurements with those of Pilgrim and Taatjes^16^ and the higher temperature shock tube data from Takahashi et al.^61^ to show that, as would be expected, reaction 1b is consistent with the negative activation energy measured for reaction −1b and that TS-1 has variational character.

To illustrate that the activation energy alone does not control the transition-state, a MESMER model using the reaction scheme given in Figure 8 was used to fit the abstraction data in Table 1, the data from Pilgrim and Taatjes^16^ and Takahashi et al.,^61^ where the TS-1 energy was floated. This analysis returned TS-1 much higher, 43 kJ mol^–1^, and the fit to the data was poor and systematically deviated strongly with temperature.

While MESMER is not capable of applying v-TST to an identified transition-state, it can be applied via an Inverse Laplace Transform (ILT) approach.^62^ A MESMER model was set up where TS-1 was removed from the scheme and replaced by an ILT reaction between the reagents and Complex-1, where the ILT parameters *A* and (*T*/298)^*n*^ were floated to fit the experimental data, and *Ea* was fixed to 23.4 kJ mol^–1^. The fit to the *k*_1b_ data is good and is shown in Figure 9, and the result is given in Table 5.

**Figure 9:**
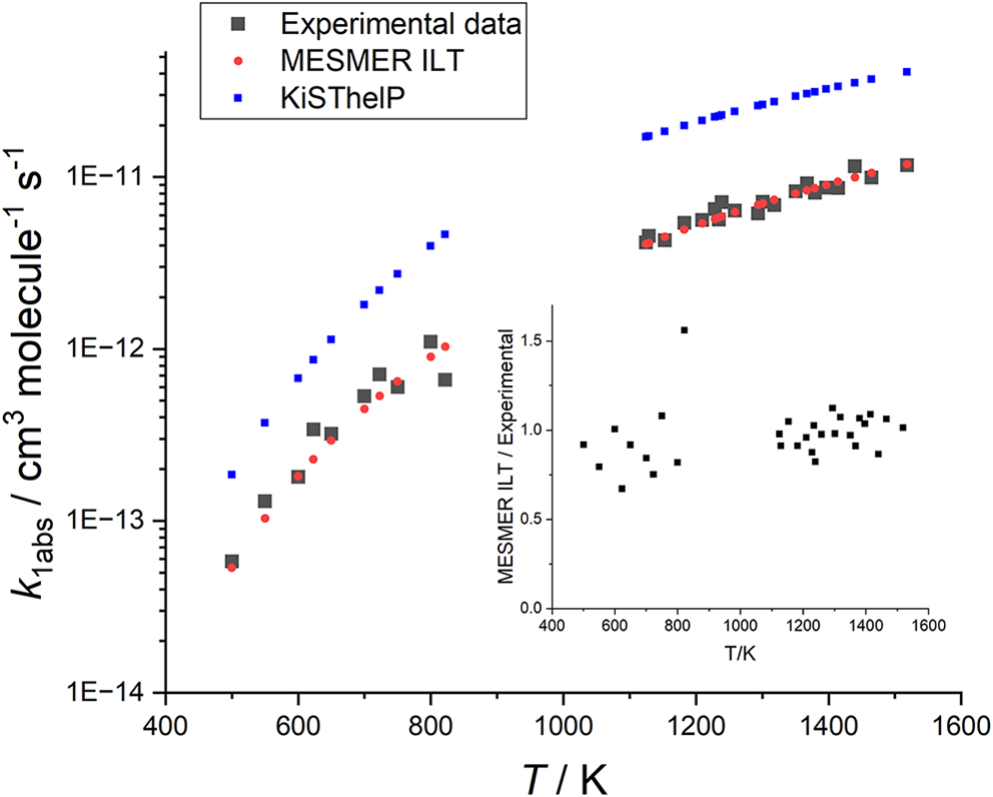
MESMER ILT fit to all *k*_1b,abs_ data, see text for details. The inset shows this data in a plot of the MESMER ILT­(red)/measured­(black) rate coefficients versus temperature; realistic errors in the data are larger than this deviation. The blue points are the result from KiSThelP, the variational transition-state calculator.

**Table 5:** MESMER ILT Abstraction Model Parameters from Fitting to the Abstraction Data from This Study, Pilgrim and Taatjes,^16^ and Takahashi et al.^61^ *k*_1b_ = *A* × (*T*/298)^*n*^ × exp­(–*E*_a_ /(*RT*))

scenario	parameters in *k*_1b_ = *A* × (*T*/298)^*n*^ × exp(–*E*_a_ /(*RT*)	values
activation energy fixed and *n* and *A* floated	*A* (cm^3^ molecule s^–1^)	(7.5 ± 2.9) × 10^–12^
	*n*	1.44 ± 0.26
	*E*_a_ (kJ mol^–1^)	23.4 fixed
	χ^*2*^/degrees of Freedom	0.93
*n* fixed to zero and *A* and *E*_a_ floated	*A* (cm^3^ molecule s^–1^)	(1.94 ± 0.25) × 10^–10^
	*n*	0 fixed
	*E_a_* (kJ mol^–1^)	35.6 ± 2.7
	χ^*2*^/degrees of Freedom	1.07

From Table 5, it is noted that these parameters imply that as the temperature increases, the transition-state becomes more reagent-like, *i.e*. the *A*-factor is increasing with *T*. A near-equal solution is obtained if *n* is fixed at 0, see Table 5. This yields an *Ea* equal to 36 kJ mol^–1^, which is close to the value reported by Takahashi et al.,^61^ where their data were simply analyzed with an Arrhenius equation. But if only the lower temperature *k*_1b_ data are considered, the smaller Arrhenius parameters of Pilgrim and Taatjes^16^ are returned. These observations are reconciled through a large positive *n* value.

While the ILT approach provides parameters from data fitting, it is not predictive and does not use calculated information along the reaction surface. *A priori* information on the rate coefficient requires v-TST. To provide insight into this abstraction reaction, variational transition state theory calculations were carried out using the software KiSThelP.^40^ The input for these calculations is provided by DKH–CCSD­(T)/CBS single point energy corrections for many points along the intrinsic reaction coordinate (IRC) mapping the direct abstraction pathway, where at each point a harmonic Hessian was calculated, and the vibrational modes orthogonal to the reaction coordinate were projected out to yield transverse frequencies suitable for inclusion in variational transition state theory calculations.^63^ This input information allows the enthalpy and entropy to be calculated at each point, which is used to identify the minimum in the free energy of activation, Δ*G* ^‡^, the transition state for a given temperature, and hence the rate coefficient. The result of this calculation is shown in Figure 9, in blue. While these calculated rate coefficients are too high, they are consistently high over the whole temperature rate, by *ca*. a factor of 4. This calculation highlights that the reaction is becoming more reagent-like as the temperature is increased, via the position of Δ*G* ^‡^. The rate coefficients given in Figure 9, in blue, are purely from calculation with no adjustment. A potential limitation in this calculation is that all the vibrations are assumed to be harmonic oscillators. This is likely to be a poor approximation for the low-frequency vibrations, which are the newly formed vibrations resulting from the reagents coming together. More sophisticated v-TST might improve the accuracy of the calculation, but the present calculation highlights the variational nature of the TS.

To further substantiate the variational nature of this abstraction TS, the reverse reaction:

R-1b $$\mathrm{HCl}+C_{2}H_{3}\overset{k_{-1b}\;}{\to }\mathrm{Cl}+C_{2}H_{4}$$

was investigated using a MESMER ILT model, see Figure 8. The above analysis of *k*_1b_ data implies that the reverse reaction rate coefficient, *k*_–1b_, will exhibit a negative temperature dependence, and that the TS will become more product-like (Cl + C_2_H_4_) with increased temperature. The literature data are sparse and are centered around room temperature. The studies by Krueger et al.^52^ and Russell et al.^53^ assigned a substantial negative *T*-dependent rate coefficient and MESMER fitting to just the Russell et al.^53^ data (a larger data set than Krueger et al.^52^) returns a value for TS-1 consistent with the energies given in Table 1 and also consistent with the values given in Table 5.

There is an additional study on *k*_–1b_ by Dobis and Benson^64^ that assigned substantially smaller rate coefficients with a positive temperature dependence. It is noted that this study is not time-resolved and more assumptions are required to assign the rate coefficients compared to the time-resolved studies by Krueger et al.^52^ and Russell et al.^53^ This study is in agreement with Krueger et al.^52^ and Russell et al.^53^

## 5 Conclusions

This study has carried out time-resolved experiments on the reaction between Cl and C_2_H_4_, R1, where the Cl atoms have been monitored for the first time via vacuum ultraviolet laser-induced fluorescence (VUV-LIF), probing the (4s ^2^D_3/2_ ← 3p ^2^P_3/2_) transition at 118.877 nm. This method of monitoring Cl has been shown to give kinetics for R1 in good agreement with those in the literature. Experimentally, the method is simpler than the four-wave mixing, laser technique used previously^6^ and more selective than resonance lamp detection. Compared to previous techniques (laser or resonance lamp) that have utilized detection at wavelengths of 134–139 nm, this method has significantly less interference from oxygen, and we have demonstrated that we can measure Cl reactivity of ambient air, allowing for accurate estimates of Cl reactivity at atmospheric pressure. The major limitation is the absorption of probe radiation at 118.877 nm by water vapor, and work is currently underway to minimize the impact of water vapor and allow measurements of Cl reactivity closer still to atmospheric pressure.

At room temperature, the kinetics of reaction R1 exhibit a strong pressure dependence and have been characterized in a significant number of previous studies. This study has carried out a limited number of measurements at room temperature, and via reaction rate theory (MESMER), it is shown that our measurements are consistent with most of the literature; parameters from this fitting are provided.

Equilibrium behavior was observed between 393 and 490 K, which is consistent with the association adduct, ClCH_2_CH_2_, redissociating to the reagents on the time scale of the forward reaction. This is the first observation of equilibrium behavior observed via monitoring Cl atoms. This equilibrium, [Disp-formula R1a]/5, has been analyzed using MESMER to fit to the forward and reverse rate coefficients, where the binding energy of Cl–C_2_H_4_ is adjusted, giving Δ_r_ *H*_R1a_^o^ = (−74.2 ± 0.6) kJ mol^–1^. A range of *ab initio* calculation methods have been used to explore reaction R1, where consistent values for Δ_r_ *H*_R1a_^o^ are returned (Δ_r_ *H*_R1a_^o^ = −76.85 to −79.80 kJ mol^–1^) but are ∼3 kJ mol^–1^ more exothermic than our experimental value. This difference suggests that these calculations are accurate to ca. 4 kJ mol^–1^, with relativistic correction improving the result by ca. 1 kJ mol^–1^.

At *T* > 500 K, R1 essentially occurs via abstraction to form HCl + C_2_H_3_, R1b. Our calculations identify a transition state, TS-1, but this is below the energy of the products, as has been observed previously in halogen + hydrocarbon reactions. MESMER fitting to R1b kinetic data from this study and the literature returns a poor fit if the model uses a fixed TS-1. This is because the transition state varies with temperature. MESMER can accommodate a variational transition state by replacing TS-1 with ILT, and such a model returns a good fit to the experimental data, see Figure 9. The ILT parameters imply that the reaction becomes reagent-like as *T* is increased. To predictively account for this variational transition state, the software KiSThelP^40^ was used to calculate R1b rate coefficients. The results highlighted the transition state, moving with temperature in accordance with the ILT parameters but overestimated the experimental data by ca. a factor of 4.

## Supplementary Material



## Notes

The MESMER model used in this study and the input files to run KiSThelP (PDF)
