# Supplementary material for: First Direct Observation of Equilibrium Involving Cl Atoms: Cl + C2H4 ⇔ ClCH2CH2 by VUV Monitoring
Source: J Phys Chem A. 2025 Oct 8;129(42):9733–44. doi: 10.1021/acs.jpca.5c05430 (PMC12557372; doi:10.1021/acs.jpca.5c05430)
Supplement: Supplementary file 1 [file jp5c05430_si_001.pdf]

First Direct Observation of Equilibrium involving Cl Atoms: Cl + C_2H_4 ⇔ $ClCH_2CH_2$ by VUV Monitoring

Mark A. Blitz*[1,2], Thomas H. Speak[3] and Paul W. Seakins[1]

1 - School of Chemistry, University of Leeds, Leeds, LS2 9JT, UK

2 – NCAS, University of Leeds, Leeds, LS2 9JT, UK

3 – University of British Columbia, Vancouver, Vancouver, V6T 1Z1, BC, Canada

**E-mail: m.blitz@leeds.ac.uk*

Supporting Information

1. **Input file for MESMER master equation analysis:** Cl + C_2H_4 Mesmer xml input

2. **Input file for KiSThelP:** C2H4-OPT-CCSDT-madef2TZVPP

3. **Input file for KiSThelP:** Cl-DKH-CCSDT-CBS-CCSDT-madef2TZVPP

3. **Input file for KiSThelP:** C2H3-H-Cl-TS-1-2-OPT-DKH-CCSDT-CBS-CCSDT-ma-def2-TZVPP-IRC

1. Cl + C_2H_4 Mesmer xml input


```
<?xml version="1.0" encoding="utf-8"?>
<?xml-stylesheet type='text/xsl' href='../../mesmer1.xsl'?>
<me:mesmer xmlns="http://www.xml-cml.org/schema"
         xmlns:me="http://www.chem.leeds.ac.uk/mesmer"
         xmlns:xsi="http://www.w3.org/2001/XMLSchema-instance">
  <me:title>Cheap Ethylene + Cl addition no barrier found </me:title>

  <me:description>
Fully harmonic model Cl C2H4 structures given are from B2PLYP-D3(BJ)/def2-TZVPP
results also from wB97X-D/def2-TZVPP and M06-2X-D3/def2-TZVPP
Single point energy corrections at the CCSD(T)/CBS level from aug-cc-pVDZ to QZ extrapolation
  </me:description>

  <moleculeList>
  <molecule id="Ethylene">

 <atomArray>
  <atom id="a1" elementType="C" x3="0.000000" y3="0.000000" z3="0.663875"/>
  <atom id="a2" elementType="H" x3="0.000000" y3="0.920778" z3="1.229675"/>
  <atom id="a3" elementType="H" x3="0.000000" y3="-0.920778" z3="1.229675"/>
  <atom id="a4" elementType="C" x3="0.000000" y3="0.000000" z3="-0.663875"/>
  <atom id="a5" elementType="H" x3="0.000000" y3="0.920778" z3="-1.229675"/>
  <atom id="a6" elementType="H" x3="0.000000" y3="-0.920778" z3="-1.229675"/>
 </atomArray>
 <bondArray>
  <bond atomRefs2="a5 a4" order="1"/>
  <bond atomRefs2="a6 a4" order="1"/>
  <bond atomRefs2="a4 a1" order="2"/>
  <bond atomRefs2="a1 a2" order="1"/>
  <bond atomRefs2="a1 a3" order="1"/>
 </bondArray>

     <propertyList>

                        <!-- ZPE corrected -->
      <property dictRef="me:ZPE">
        <scalar units="kJ/mol">0.0</scalar>
      </property>

                       <property dictRef="me:frequenciesScaleFactor">
                       <!-- anharmonic -->
         <scalar>1.0</scalar>
      </property>
    <property title="Vibrational Frequencies" dictRef="me:vibFreqs">
```

```
<array units="cm-1">3261.908 3156.482 1488.625 837.545 1069.852 983.426 3234.166 3171.898 1689.585 1386.335 1253.003
974.095</array>

</property>
  <property title="Rotational Constants" dictRef="me:rotConsts">
    <!-- B2PLYP-D3BJ def2TZVPP HO -->
  <!-- <array units="cm-1">4.929 1.010 0.838 </array> -->
    <!-- B2PLYP-D3BJ def2TZVPP anharm -->
    <array units="cm-1">4.872010 1.006279 0.830434</array>
</property>


<property title="Symmetry Number" dictRef="me:symmetryNumber">
 <scalar>4</scalar>
</property>
     <property dictRef="me:MW">
       <scalar>28.0313</scalar>
     </property>
     <property dictRef="me:spinMultiplicity">
       <scalar>1</scalar>
     </property>
     <property dictRef="me:sigma">
       <scalar>3.79</scalar>
     </property>
     <property dictRef="me:epsilon">
       <scalar>153.0</scalar>
     </property>
   </propertyList>

   <me:energyTransferModel xsi:type="me:ExponentialDown">
     <!-- <me:deltaEDown bathGas="He" units="cm-1" lower="50.43" upper="57.97" stepsize="0.10" >54.19</me:deltaEDown> -->
       <!-- <me:deltaEDownTExponent bathGas="He" referenceTemperature="298" lower="1.1141" upper="1.235" stepsize="0.01">1.1749</me:deltaEDownTExponent> -->
     <!-- <me:deltaEDown bathGas="He" units="cm-1" lower="30" upper="250" stepsize="0.10" >65.45</me:deltaEDown> -->
       <!-- <me:deltaEDownTExponent bathGas="He" referenceTemperature="298" lower="-1.35" upper="1.35" stepsize="0.01">1.20</me:deltaEDownTExponent> -->
         <me:deltaEDown bathGas="N2" units="cm-1" lower="65" upper="450" stepsize="0.10" >200</me:deltaEDown>
       <me:deltaEDownTExponent bathGas="N2" referenceTemperature="298">0.25</me:deltaEDownTExponent>
     <!-- <me:deltaEDownTExponent bathGas="N2" referenceTemperature="298" lower="-1.35" upper="1.45" stepsize="0.01">0.25</me:deltaEDownTExponent> -->
         <me:deltaEDown bathGas="Ar" units="cm-1" lower="65" upper="250" stepsize="0.10" >143</me:deltaEDown>
       <!-- <me:deltaEDownTExponent bathGas="Ar" referenceTemperature="298">1.00</me:deltaEDownTExponent> -->
     <me:deltaEDownTExponent bathGas="Ar" referenceTemperature="298" lower="-1.35" upper="1.35" stepsize="0.01">1.0</me:deltaEDownTExponent>
    </me:energyTransferModel>

  <me:DOSCMethod xsi:type="me:QMRotors"/>

    </molecule>


<molecule id="Cl">
   <atom elementType="Cl"/>
   <propertyList>

     <!-- ZPE corrected -->
     <property dictRef="me:ZPE">
       <scalar units="kJ/mol">0.0</scalar>
     </property>
     <!-- ZPE uncorrected -->
     <!-- CCSD(T) CBS corrected B2PLYP D3BJ def2TZVPP  -->
       <!-- <property dictRef="me:ZPE" zeroPointVibEnergyAdded="false"> -->
       <!-- <scalar units="Hartree" convention="computational">-459.7050503</scalar> -->
     <!-- </property> -->

     <property dictRef="me:MW">
       <scalar>34.96885</scalar>
     </property>
     <property dictRef="me:spinMultiplicity">
       <scalar>2</scalar>
     </property>

       <property dictRef="me:eletronicExcitation">
       <array units="cm-1">881</array>
     </property>

       <!-- I have not changed this from the H ethylene file -->
     <property dictRef="me:sigma">
       <scalar>2.00</scalar>
     </property>
     <property dictRef="me:epsilon">
       <scalar>153.0</scalar>
     </property>

   </propertyList>

   <me:energyTransferModel xsi:type="me:ExponentialDown">
     <!-- <me:deltaEDown bathGas="He" units="cm-1" lower="50.43" upper="57.97" stepsize="0.10" >54.19</me:deltaEDown> -->
       <!-- <me:deltaEDownTExponent bathGas="He" referenceTemperature="298" lower="1.1141" upper="1.235" stepsize="0.01">1.1749</me:deltaEDownTExponent> -->

     <!-- <me:deltaEDown bathGas="He" units="cm-1" lower="30" upper="250" stepsize="0.10" >65.45</me:deltaEDown> -->
       <!-- <me:deltaEDownTExponent bathGas="He" referenceTemperature="298" lower="-1.35" upper="1.35" stepsize="0.01">1.20</me:deltaEDownTExponent> -->

         <me:deltaEDown bathGas="N2" units="cm-1" lower="65" upper="450" stepsize="0.10" >200</me:deltaEDown>
       <me:deltaEDownTExponent bathGas="N2" referenceTemperature="298">0.25</me:deltaEDownTExponent>
     <!-- <me:deltaEDownTExponent bathGas="N2" referenceTemperature="298" lower="-1.35" upper="1.45" stepsize="0.01">0.25</me:deltaEDownTExponent> -->

         <me:deltaEDown bathGas="Ar" units="cm-1" lower="65" upper="250" stepsize="0.10" >143</me:deltaEDown>
       <!-- <me:deltaEDownTExponent bathGas="Ar" referenceTemperature="298">1.00</me:deltaEDownTExponent> -->
     <me:deltaEDownTExponent bathGas="Ar" referenceTemperature="298" lower="-1.35" upper="1.35" stepsize="0.01">1.0</me:deltaEDownTExponent>
    </me:energyTransferModel>

  <me:DOSCMethod xsi:type="me:QMRotors"/>
  </molecule>

<molecule id="C2H4Cl" spinMultiplicity="2">

<atomArray>
 <atom id="a1" elementType="C" x3="0.582338" y3="0.654952" z3="0.000002"/>
 <atom id="a2" elementType="Cl" x3="-1.079929" y3="-0.158859" z3="-0.000001"/>
 <atom id="a3" elementType="H" x3="0.580011" y3="1.273016" z3="-0.889989"/>
 <atom id="a4" elementType="H" x3="0.580010" y3="1.273011" z3="0.889997"/>
 <atom id="a5" elementType="C" spinMultiplicity="2" x3="1.634114" y3="-0.358756" z3="0.000000"/>
 <atom id="a6" elementType="H" x3="1.950036" y3="-0.811290" z3="-0.925947"/>
 <atom id="a7" elementType="H" x3="1.950017" y3="-0.811312" z3="0.925942"/>
</atomArray>
<bondArray>
 <bond id="b1" atomRefs2="a6 a5" order="1"/>
 <bond id="b2"  atomRefs2="a3 a1" order="1"/>
 <bond id="b3" atomRefs2="a2 a1" order="1"/>
 <bond id="b4" atomRefs2="a5 a1" order="1"/>
 <bond id="b5" atomRefs2="a5 a7" order="1"/>
 <bond id="b6" atomRefs2="a1 a4" order="1"/>
</bondArray>
   <propertyList>

     <!-- ZPE corrected -->
    <property dictRef="me:ZPE">
    <scalar units="kJ/mol" lower="-95.4" upper="-65.4" stepsize="0.01">-75.4</scalar>
     </property>

     <property dictRef="me:frequenciesScaleFactor">
     <!-- anharmonic -->
       <scalar>1.0</scalar>

     </property>
   <property title="Vibrational Frequencies" dictRef="me:vibFreqs">
<array units="cm-1">3152.08 3036.486 3063.987 3010.298 1475.216 1454.246 1235.132 1231.185 1083.546 1048.475 783.457 662.767
546.89 305.245 </array>
</property>

  <property title="Rotational Constants" dictRef="me:rotConsts">
  <array units="cm-1">1.081837 0.190370 0.172877</array>
 </property>
```

```
    <property dictRef="me:MW">
      <scalar>63.00017</scalar>
    </property>

                                                            <property dictRef="me:spinMultiplicity">
      <scalar>2</scalar>
                                                            <!-- <scalar>2</scalar> -->
    </property>

                                                           <property title="Symmetry Number" dictRef="me:symmetryNumber">
                                                           <!-- This is what I see correct if you need to -->
                                                          <scalar>2</scalar>
                                                          </property>

                                                          <!-- values from the H ethylene ethyl  -->
     <property dictRef="me:sigma">
     <scalar>3.79</scalar>
    </property>
    <property dictRef="me:epsilon">
      <scalar>153.0</scalar>
    </property>
  </propertyList>

  <me:energyTransferModel xsi:type="me:ExponentialDown">
                              <me:deltaEDown bathGas="He" units="cm-1" lower="50.43" upper="250" stepsize="0.10" >100</me:deltaEDown>
                                                <me:deltaEDownTExponent bathGas="He" referenceTemperature="298">1.00</me:deltaEDownTExponent>
                                                  <!-- <me:deltaEDownTExponent bathGas="He" referenceTemperature="298" lower="1.1141" upper="1.235" stepsize="0.01">1.1749</me:deltaEDownTExponent> -->

                              <!-- <me:deltaEDown bathGas="He" units="cm-1" lower="30" upper="250" stepsize="0.10" >65.45</me:deltaEDown> -->
                                                  <!-- <me:deltaEDownTExponent bathGas="He" referenceTemperature="298" lower="-1.35" upper="1.35" stepsize="0.01">1.20</me:deltaEDownTExponent> -->

                                                                   <me:deltaEDown bathGas="N2" units="cm-1" lower="65" upper="450" stepsize="0.10" >200</me:deltaEDown>
                                                  <me:deltaEDownTExponent bathGas="N2" referenceTemperature="298">0.25</me:deltaEDownTExponent>
                               <!-- <me:deltaEDownTExponent bathGas="N2" referenceTemperature="298" lower="-1.35" upper="1.45" stepsize="0.01">0.25</me:deltaEDownTExponent> -->

                                                                  <me:deltaEDown bathGas="Ar" units="cm-1" lower="65" upper="250" stepsize="0.10" >173</me:deltaEDown>
                                                  <!-- <me:deltaEDownTExponent bathGas="Ar" referenceTemperature="298">1.49</me:deltaEDownTExponent> -->
                               <me:deltaEDownTExponent bathGas="Ar" referenceTemperature="298" lower="-1.35" upper="1.45" stepsize="0.01">0.75</me:deltaEDownTExponent>
  </me:energyTransferModel>

<me:ExtraDOSCMethod xsi:type="me:HinderedRotorQM1D">
    <me:bondRef>b4</me:bondRef>
    <me:HinderedRotorPotential format="analytical" units="kJ/mol">
      <me:PotentialPoint index="0"   coefficient="4.5"/>
      <me:PotentialPoint index="3"  coefficient="-4.5"/>
                                                         <!-- <me:PotentialPoint index="0"   coefficient="4.5"/> -->
      <!-- <me:PotentialPoint index="3"  coefficient="-4.5"/> -->
    </me:HinderedRotorPotential>
    <me:periodicity>1</me:periodicity>
  </me:ExtraDOSCMethod>
<me:DOSCMethod xsi:type="me:QMRotors"/>

  </molecule>

  <molecule id="He">
   <atom elementType="He"/>
   <propertyList>
    <property dictRef="me:epsilon">
      <scalar>10.22</scalar>
    </property>
    <property dictRef="me:sigma">
      <scalar>2.511</scalar>
    </property>
    <property dictRef="me:MW">
      <scalar>4.04</scalar>
    </property>
   </propertyList>
                             <me:DOSCMethod xsi:type="me:QMRotors"/>
  </molecule>


                           <molecule id="Ar">
   <atom elementType="Ar"/>
   <propertyList>
    <property dictRef="me:epsilon">
      <scalar>114</scalar>
    </property>
    <property dictRef="me:sigma">
      <scalar>3.47</scalar>
    </property>
    <property dictRef="me:MW">
      <scalar>39.948</scalar>
    </property>
                                                          <property dictRef="me:spinMultiplicity">
      <scalar>1</scalar>
    </property>
   </propertyList>
                             <me:DOSCMethod xsi:type="me:QMRotors"/>
                            <!-- <me:DOSCMethod>ClassicalRotors</me:DOSCMethod>   -->
  </molecule>

                            <molecule id="N2">
   <atom elementType="N"/>
   <propertyList>
    <property dictRef="me:epsilon">
      <scalar>48.0</scalar>
    </property>
    <property dictRef="me:sigma">
      <scalar>3.90</scalar>
    </property>
    <property dictRef="me:MW">
      <scalar units="amu">28.0</scalar>
    </property>
                                                          <property dictRef="me:spinMultiplicity">
      <scalar>1</scalar>
    </property>
   </propertyList>
                             <me:DOSCMethod xsi:type="me:QMRotors"/>
                          <!-- <me:DOSCMethod>ClassicalRotors</me:DOSCMethod>   -->
  </molecule>



</moleculeList>

<reactionList>

  <reaction id="R1" reversible="true">
   <reactant>
    <molecule ref="Ethylene"  role="excessReactant" />
   </reactant>

   <reactant>
    <molecule ref="Cl"  role="deficientReactant" />
   </reactant>

   <product>
    <molecule ref="C2H4Cl"  role="modelled" />
   </product>

                             <me:MCRCMethod xsi:type="me:MesmerILT">

                              <!-- <me:preExponential >2.70e-10</me:preExponential> -->
                             <me:preExponential lower="1e-13" upper="9e-10" stepsize="1e-13">2.3e-10</me:preExponential>
                                                 <!-- <me:activationEnergy units="kJ/mol" lower="0.0" upper="20.0" stepsize="0.01">0.00</me:activationEnergy> -->
    <!-- <me:TInfinity>298.0</me:TInfinity> -->
                                                 <!-- <me:nInfinity lower="-1.0" upper="1.0" stepsize="0.005">-0.25</me:nInfinity> -->

                                                <me:activationEnergy units="kJ/mol" >0</me:activationEnergy>
```

<me:TInfinity>298.0</me:TInfinity>
<me:nInfinity >-0.2</me:nInfinity>

</me:MCRCMethod>
<me:excessReactantConc>1e13</me:excessReactantConc>

</reaction>

</reactionList>

<me:conditions>
<me:bathGas>Ar</me:bathGas>
<me:PTs>

<!-- 6thMay more N2 with 213nm -->
<me:PTpair units="Torr" P= "9.4" T= "295" precision="dd"> <me:bathGas>Ar</me:bathGas> <me:experimentalRate ref1="Cl" ref2="Cl" refReaction="R1" error="2.70E-13">2.70E-12 </me:experimentalRate> </me:PTpair>
<me:PTpair units="Torr" P= "9.4" T= "295" precision="dd"> <me:bathGas>N2</me:bathGas> <me:experimentalRate ref1="Cl" ref2="Cl" refReaction="R1" error="3.30E-13">3.30E-12 </me:experimentalRate> </me:PTpair>
<me:PTpair units="Torr" P= "37" T= "295" precision="dd"> <me:bathGas>N2</me:bathGas> <me:experimentalRate ref1="Cl" ref2="Cl" refReaction="R1" error="1.08E-12">1.08E-11 </me:experimentalRate> </me:PTpair>
<me:PTpair units="Torr" P= "119.9" T= "295" precision="dd"> <me:bathGas>N2</me:bathGas> <me:experimentalRate ref1="Cl" ref2="Cl" refReaction="R1" error="2.78E-12">2.78E-11 </me:experimentalRate> </me:PTpair>

<!-- 11th April -->
<!-- 266 nm -->
<me:PTpair units="Torr" P= "9.2" T= "295" precision="dd"> <me:bathGas>N2</me:bathGas> <me:experimentalRate ref1="Cl" ref2="Cl" refReaction="R1" error="2.85E-13">2.86E-12 </me:experimentalRate> </me:PTpair>
<me:PTpair units="Torr" P= "31.2" T= "295" precision="dd"> <me:bathGas>N2</me:bathGas> <me:experimentalRate ref1="Cl" ref2="Cl" refReaction="R1" error="8E-13">7.98E-12 </me:experimentalRate>
</me:PTpair>
<me:PTpair units="Torr" P= "87.8" T= "295" precision="dd"> <me:bathGas>N2</me:bathGas> <me:experimentalRate ref1="Cl" ref2="Cl" refReaction="R1" error="2E-12">2.01E-11 </me:experimentalRate>
</me:PTpair>
<me:PTpair units="Torr" P= "297" T= "295" precision="dd"> <me:bathGas>N2</me:bathGas> <me:experimentalRate ref1="Cl" ref2="Cl" refReaction="R1" error="5.0E-12">4.77E-11
</me:experimentalRate> </me:PTpair>

<!-- 25th MArch RT -->

<me:PTpair units="Torr" P= "31.9" T= "295" precision="dd"> <me:bathGas>Ar</me:bathGas> <me:experimentalRate ref1="Cl" ref2="Cl" refReaction="R1" error="6.8E-13">6.82E-12 </me:experimentalRate> </me:PTpair>
<me:PTpair units="Torr" P= "73" T= "295" precision="dd"> <me:bathGas>Ar</me:bathGas> <me:experimentalRate ref1="Cl" ref2="Cl" refReaction="R1" error="1.18E-12">1.182E-11 </me:experimentalRate>
</me:PTpair>
<me:PTpair units="Torr" P= "143" T= "295" precision="dd"> <me:bathGas>Ar</me:bathGas> <me:experimentalRate ref1="Cl" ref2="Cl" refReaction="R1" error="2.46E-12">2.462E-11
</me:experimentalRate> </me:PTpair>
<me:PTpair units="Torr" P= "298" T= "295" precision="dd"> <me:bathGas>Ar</me:bathGas> <me:experimentalRate ref1="Cl" ref2="Cl" refReaction="R1" error="3.24E-12">3.242E-11 </me:experimentalRate> </me:PTpair>
<!-- 27th March -->
<me:PTpair units="Torr" P= "36.4" T= "393" precision="dd"> <me:bathGas>Ar</me:bathGas> <me:experimentalRate ref1="Cl" ref2="Cl" refReaction="R1" error="3.1E-13">3.13E-12 </me:experimentalRate>
</me:PTpair>

<!-- <me:PTpair units="Torr" P= "36.3" T= "490" precision="dd"> <me:bathGas>Ar</me:bathGas> <me:experimentalRate ref1="Cl" ref2="Cl" refReaction="R1" error="3.2E-13">7.62E-
13</me:experimentalRate> </me:PTpair> -->
<!-- <me:PTpair units="Torr" P= "36.3" T= "490" precision="dd"> <me:bathGas>Ar</me:bathGas> <me:experimentalRate ref1="Cl" ref2="Cl" refReaction="R1" error="3.2E-13">8.27E-
13</me:experimentalRate> </me:PTpair> -->

<!-- 26thMarch -->
<me:PTpair units="Torr" P= "36.2" T= "417" precision="dd"> <me:bathGas>Ar</me:bathGas> <me:experimentalRate ref1="Cl" ref2="Cl" refReaction="R1" error="2.4E-13">2.42E-12 </me:experimentalRate> </me:PTpair>
<me:PTpair units="Torr" P= "36.4" T= "441" precision="dd"> <me:bathGas>Ar</me:bathGas> <me:experimentalRate ref1="Cl" ref2="Cl" refReaction="R1" error="1.9E-13">1.95E-12 </me:experimentalRate> </me:PTpair>

<me:PTpair units="Torr" P= "36.2" T= "465" precision="dd"> <me:bathGas>Ar</me:bathGas> <me:experimentalRate ref1="Cl" ref2="Cl" refReaction="R1" error="1.5E-13">1.48E-12</me:experimentalRate> </me:PTpair>

<!-- 2nd April -->
<!-- <me:PTpair units="Torr" P= "35" T= "490" precision="dd"> <me:bathGas>Ar</me:bathGas> <me:experimentalRate ref1="Cl" ref2="Cl" refReaction="R1" error="1.8E-13">7.81E-
13</me:experimentalRate> </me:PTpair> -->

<me:PTpair units="Torr" P= "98.8" T= "409" precision="dd"> <me:bathGas>Ar</me:bathGas> <me:experimentalRate ref1="Cl" ref2="Cl" refReaction="R1" error="7.1E-
13">7.09E-12 </me:experimentalRate> </me:PTpair>
<me:PTpair units="Torr" P= "99.3" T= "430" precision="dd"> <me:bathGas>Ar</me:bathGas> <me:experimentalRate ref1="Cl" ref2="Cl" refReaction="R1" error="5.2E-
13">5.21E-12 </me:experimentalRate> </me:PTpair>
<me:PTpair units="Torr" P= "99.5" T= "452" precision="dd"> <me:bathGas>Ar</me:bathGas> <me:experimentalRate ref1="Cl" ref2="Cl" refReaction="R1" error="3.7E-
13">3.72E-12</me:experimentalRate> </me:PTpair>
<me:PTpair units="Torr" P= "99.3" T= "473" precision="dd"> <me:bathGas>Ar</me:bathGas> <me:experimentalRate ref1="Cl" ref2="Cl"
refReaction="R1" error="3.4E-13">3.37E-12</me:experimentalRate> </me:PTpair>

<me:PTpair units="Torr" P= "100.2" T= "430" precision="dd"> <me:bathGas>N2</me:bathGas> <me:experimentalRate ref1="Cl" ref2="Cl" refReaction="R1" error="6.3E-
13">6.28E-12 </me:experimentalRate> </me:PTpair>

<!-- 10thApril -->

<me:PTpair units="Torr" P= "49.8" T= "473" precision="dd"> <me:bathGas>He</me:bathGas> <me:experimentalRate ref1="Cl" ref2="Cl" refReaction="R1" error="2.1E-13">2.06E-12 </me:experimentalRate>
</me:PTpair>
<me:PTpair units="Torr" P= "49.7" T= "423" precision="dd"> <me:bathGas>He</me:bathGas> <me:experimentalRate ref1="Cl" ref2="Cl" refReaction="R1" error="2.6E-13">2.57E-12 </me:experimentalRate>
</me:PTpair>

<!-- 27th MArch -->
<me:PTpair units="Torr" P= "36.4" T= "393" precision="dd"> <me:bathGas>Ar</me:bathGas> <me:experimentalRate ref1="C2H4Cl" ref2="C2H4Cl" refReaction="R1" error="16">157</me:experimentalRate> </me:PTpair>

<!-- <me:PTpair units="Torr" P= "36.3" T= "490" precision="dd"> <me:bathGas>Ar</me:bathGas> <me:experimentalRate ref1="C2H4Cl" ref2="C2H4Cl" refReaction="R1" error="3640">13095</me:experimentalRate> </me:PTpair> -->
<!-- <me:PTpair units="Torr" P= "36.3" T= "490" precision="dd"> <me:bathGas>Ar</me:bathGas> <me:experimentalRate ref1="C2H4Cl" ref2="C2H4Cl" refReaction="R1" error="3640">11734</me:experimentalRate> </me:PTpair> -->

<!-- 26th March -->
<me:PTpair units="Torr" P= "36.2" T= "417" precision="dd"> <me:bathGas>Ar</me:bathGas> <me:experimentalRate ref1="C2H4Cl" ref2="C2H4Cl" refReaction="R1" error="56">564</me:experimentalRate> </me:PTpair>
<me:PTpair units="Torr" P= "36.4" T= "441" precision="dd"> <me:bathGas>Ar</me:bathGas> <me:experimentalRate ref1="C2H4Cl" ref2="C2H4Cl" refReaction="R1" error="178">1775</me:experimentalRate> </me:PTpair>

<me:PTpair units="Torr" P= "35" T= "465" precision="dd"> <me:bathGas>Ar</me:bathGas> <me:experimentalRate ref1="C2H4Cl" ref2="C2H4Cl" refReaction="R1" error="375">3748</me:experimentalRate> </me:PTpair>

<!-- 2ndArpil -->
<!-- <me:PTpair units="Torr" P= "35" T= "490" precision="dd"> <me:bathGas>Ar</me:bathGas> <me:experimentalRate ref1="C2H4Cl" ref2="C2H4Cl" refReaction="R1" error="1600">8967</me:experimentalRate> </me:PTpair> -->

<!-- 10thApril -->

<me:PTpair units="Torr" P= "50" T= "473" precision="dd"> <me:bathGas>He</me:bathGas> <me:experimentalRate ref1="C2H4Cl" ref2="C2H4Cl" refReaction="R1" error="560">5600</me:experimentalRate> </me:PTpair>
<me:PTpair units="Torr" P= "50" T= "423" precision="dd"> <me:bathGas>He</me:bathGas> <me:experimentalRate ref1="C2H4Cl" ref2="C2H4Cl" refReaction="R1" error="76">759</me:experimentalRate> </me:PTpair>

<!-- 10% ERRORS -->
<!-- 8thApril -->

<me:PTpair units="Torr" P= "98.8" T= "409" precision="dd"> <me:bathGas>Ar</me:bathGas> <me:experimentalRate ref1="C2H4Cl" ref2="C2H4Cl" refReaction="R1" error="76">761</me:experimentalRate> </me:PTpair>
<me:PTpair units="Torr" P= "99.3" T= "430" precision="dd"> <me:bathGas>Ar</me:bathGas> <me:experimentalRate ref1="C2H4Cl" ref2="C2H4Cl" refReaction="R1" error="212">2119</me:experimentalRate> </me:PTpair>

<me:PTpair units="Torr" P= "99.5" T= "452" precision="dd"> <me:bathGas>Ar</me:bathGas> <me:experimentalRate ref1="C2H4Cl" ref2="C2H4Cl" refReaction="R1" error="529">5292</me:experimentalRate> </me:PTpair>
<me:PTpair units="Torr" P= "99.3" T= "472" precision="dd"> <me:bathGas>Ar</me:bathGas> <me:experimentalRate ref1="C2H4Cl" ref2="C2H4Cl" refReaction="R1" error="1060">10848</me:experimentalRate> </me:PTpair>

<me:PTpair units="Torr" P= "100.2" T= "430" precision="dd"> <me:bathGas>N2</me:bathGas> <me:experimentalRate ref1="C2H4Cl" ref2="C2H4Cl" refReaction="R1" error="220">2186</me:experimentalRate> </me:PTpair>

<!-- LITERATURE -->

<!-- COQUET 2002 -->

<me:PTpair units="Torr" P= "760" T= "295" precision="dd"> <me:bathGas>N2</me:bathGas> <me:experimentalRate ref1="Cl" ref2="Cl" refReaction="R1" error="1.1e-11">9.9e-11</me:experimentalRate> </me:PTpair>

<!-- ATKINSON 1985 -->
<me:PTpair units="Torr" P= "760" T= "295" precision="dd"> <me:bathGas>N2</me:bathGas> <me:experimentalRate ref1="Cl" ref2="Cl" refReaction="R1" error="1.2e-11">11.7e-11</me:experimentalRate> </me:PTpair>

<!-- Kaiser 1996 -->
<me:PTpair units="Torr" P= "760" T= "295" precision="dd"> <me:bathGas>N2</me:bathGas> <me:experimentalRate ref1="Cl" ref2="Cl" refReaction="R1" error="1.0e-11">10.0e-11</me:experimentalRate> </me:PTpair>

<!-- coquet 2000 -->
<me:PTpair units="Torr" P= "760" T= "295" precision="dd"> <me:bathGas>N2</me:bathGas> <me:experimentalRate ref1="Cl" ref2="Cl" refReaction="R1" error="1.2e-11">9.3e-11</me:experimentalRate> </me:PTpair>

<!-- Iwasaki 2010 -->

<me:PTpair units="Torr" P= "5" T= "295" precision="dd"> <me:bathGas>N2</me:bathGas> <me:experimentalRate ref1="Cl" ref2="Cl" refReaction="R1" error="0.40e-12">2.54e-12</me:experimentalRate> </me:PTpair>
<me:PTpair units="Torr" P= "10" T= "295" precision="dd"> <me:bathGas>N2</me:bathGas> <me:experimentalRate ref1="Cl" ref2="Cl" refReaction="R1" error="0.81e-12">3.96e-12</me:experimentalRate>
</me:PTpair>
<me:PTpair units="Torr" P= "20" T= "295" precision="dd"> <me:bathGas>N2</me:bathGas> <me:experimentalRate ref1="Cl" ref2="Cl" refReaction="R1" error="1.14e-12">7.64e-12</me:experimentalRate>
</me:PTpair>

<!-- Glowacki 2007 -->

<!-- WAllINGTON FROM GRAPH -->
<!-- THIS IS ETHYLENE -->

<me:PTpair units="Torr" P=" 0.207968373 " T= " 295 " precision="dd"> <me:bathGas>N2</me:bathGas>
<me:experimentalRate ref1="Cl" ref2="Cl" refReaction="R1" error= " 1.33E-14 " > 1.33E-13
</me:experimentalRate> </me:PTpair>
<me:PTpair units="Torr" P=" 0.311824827 " T= " 295 " precision="dd"> <me:bathGas>N2</me:bathGas>
<me:experimentalRate ref1="Cl" ref2="Cl" refReaction="R1" error= " 1.79E-14 " > 1.79E-13
</me:experimentalRate> </me:PTpair>
<me:PTpair units="Torr" P=" 0.554943776 " T= " 295 " precision="dd"> <me:bathGas>N2</me:bathGas>
<me:experimentalRate ref1="Cl" ref2="Cl" refReaction="R1" error= " 2.67E-14 " > 2.67E-13
</me:experimentalRate> </me:PTpair>
<me:PTpair units="Torr" P=" 0.669021051 " T= " 295 " precision="dd"> <me:bathGas>N2</me:bathGas>
<me:experimentalRate ref1="Cl" ref2="Cl" refReaction="R1" error= " 3.34E-14 " > 3.34E-13
</me:experimentalRate> </me:PTpair>
<me:PTpair units="Torr" P=" 0.987614096 " T= " 295 " precision="dd"> <me:bathGas>N2</me:bathGas>
<me:experimentalRate ref1="Cl" ref2="Cl" refReaction="R1" error= " 4.54E-14 " > 4.54E-13
</me:experimentalRate> </me:PTpair>
<me:PTpair units="Torr" P=" 1.527680293 " T= " 295 " precision="dd"> <me:bathGas>N2</me:bathGas>
<me:experimentalRate ref1="Cl" ref2="Cl" refReaction="R1" error= " 7.05E-14 " > 7.05E-13
</me:experimentalRate> </me:PTpair>
<me:PTpair units="Torr" P=" 3.770983815 " T= " 295 " precision="dd"> <me:bathGas>N2</me:bathGas>
<me:experimentalRate ref1="Cl" ref2="Cl" refReaction="R1" error= " 1.57E-13 " > 1.57E-12
</me:experimentalRate> </me:PTpair>
<me:PTpair units="Torr" P=" 9.308439076 " T= " 295 " precision="dd"> <me:bathGas>N2</me:bathGas>
<me:experimentalRate ref1="Cl" ref2="Cl" refReaction="R1" error= " 3.68E-13 " > 3.68E-12
</me:experimentalRate> </me:PTpair>
<me:PTpair units="Torr" P=" 24.83868227 " T= " 295 " precision="dd"> <me:bathGas>N2</me:bathGas>
<me:experimentalRate ref1="Cl" ref2="Cl" refReaction="R1" error= " 8.06E-13 " > 8.06E-12
</me:experimentalRate> </me:PTpair>
<me:PTpair units="Torr" P=" 48.53580603 " T= " 295 " precision="dd"> <me:bathGas>N2</me:bathGas>
<me:experimentalRate ref1="Cl" ref2="Cl" refReaction="R1" error= " 1.49E-12 " > 1.49E-11
</me:experimentalRate> </me:PTpair>
<me:PTpair units="Torr" P=" 93.37487654 " T= " 295 " precision="dd"> <me:bathGas>N2</me:bathGas>
<me:experimentalRate ref1="Cl" ref2="Cl" refReaction="R1" error= " 2.50E-12 " > 2.50E-11
</me:experimentalRate> </me:PTpair>
<me:PTpair units="Torr" P=" 194.1902113 " T= " 295 " precision="dd"> <me:bathGas>N2</me:bathGas>
<me:experimentalRate ref1="Cl" ref2="Cl" refReaction="R1" error= " 4.04E-12 " > 4.04E-11
</me:experimentalRate> </me:PTpair>
<me:PTpair units="Torr" P=" 286.6651052 " T= " 295 " precision="dd"> <me:bathGas>N2</me:bathGas>
<me:experimentalRate ref1="Cl" ref2="Cl" refReaction="R1" error= " 7.16E-12 " > 7.16E-11
</me:experimentalRate> </me:PTpair>
<me:PTpair units="Torr" P=" 486.8727032 " T= " 295 " precision="dd"> <me:bathGas>N2</me:bathGas>
<me:experimentalRate ref1="Cl" ref2="Cl" refReaction="R1" error= " 9.10E-12 " > 9.10E-11
</me:experimentalRate> </me:PTpair>
<me:PTpair units="Torr" P=" 696.6764387 " T= " 295 " precision="dd"> <me:bathGas>N2</me:bathGas>
<me:experimentalRate ref1="Cl" ref2="Cl" refReaction="R1" error= " 9.47E-12 " > 9.47E-11
</me:experimentalRate> </me:PTpair>
<me:PTpair units="Torr" P=" 1471.615375 " T= " 295 " precision="dd"> <me:bathGas>N2</me:bathGas>
<me:experimentalRate ref1="Cl" ref2="Cl" refReaction="R1" error= " 1.45E-11 " > 1.45E-10
</me:experimentalRate> </me:PTpair>
<me:PTpair units="Torr" P=" 2206.519212 " T= " 295 " precision="dd"> <me:bathGas>N2</me:bathGas>
<me:experimentalRate ref1="Cl" ref2="Cl" refReaction="R1" error= " 1.51E-11 " > 1.51E-10
</me:experimentalRate> </me:PTpair>
<me:PTpair units="Torr" P=" 2920.746808 " T= " 295 " precision="dd"> <me:bathGas>N2</me:bathGas>
<me:experimentalRate ref1="Cl" ref2="Cl" refReaction="R1" error= " 1.79E-11 " > 1.79E-10
</me:experimentalRate> </me:PTpair>

<!-- GLOWACKI FROM GRAPH -->

<!-- <me:PTpair units="PPCC" P=" 3.5503E+17" T="295 " precision="dd"> <me:bathGas>N2</me:bathGas> <me:experimentalRate ref1="Cl" ref2="Cl"
refReaction="R1" error= " 1.02E-12 " > 6.83E-12 </me:experimentalRate> </me:PTpair> -->
<!-- <me:PTpair units="PPCC" P=" 7.69231E+17" T="295 " precision="dd"> <me:bathGas>N2</me:bathGas> <me:experimentalRate ref1="Cl" ref2="Cl"
refReaction="R1" error= " 1.61E-12 " > 1.08E-11 </me:experimentalRate> </me:PTpair> -->
<!-- <me:PTpair units="PPCC" P=" 1.2426E+18" T="295 " precision="dd"> <me:bathGas>N2</me:bathGas> <me:experimentalRate ref1="Cl" ref2="Cl"
refReaction="R1" error= " 3.07E-12 " > 2.05E-11 </me:experimentalRate> </me:PTpair> -->
<!-- <me:PTpair units="PPCC" P=" 1.12426E+18" T="295 " precision="dd"> <me:bathGas>N2</me:bathGas> <me:experimentalRate ref1="Cl" ref2="Cl"
refReaction="R1" error= " 2.45E-12 " > 1.63E-11 </me:experimentalRate> </me:PTpair> -->
<!-- <me:PTpair units="PPCC" P=" 1.47929E+18" T="295 " precision="dd"> <me:bathGas>N2</me:bathGas> <me:experimentalRate ref1="Cl" ref2="Cl"
refReaction="R1" error= " 2.54E-12 " > 1.70E-11 </me:experimentalRate> </me:PTpair> -->
<!-- <me:PTpair units="PPCC" P=" 2.95858E+18" T="295 " precision="dd"> <me:bathGas>N2</me:bathGas> <me:experimentalRate ref1="Cl" ref2="Cl"
refReaction="R1" error= " 3.76E-12 " > 2.50E-11 </me:experimentalRate> </me:PTpair> -->
<!-- <me:PTpair units="PPCC" P=" 2.39645E+18" T="295 " precision="dd"> <me:bathGas>N2</me:bathGas> <me:experimentalRate ref1="Cl" ref2="Cl"
refReaction="R1" error= " 3.35E-12 " > 2.23E-11 </me:experimentalRate> </me:PTpair> -->
<!-- <me:PTpair units="PPCC" P=" 2.39645E+18" T="295 " precision="dd"> <me:bathGas>N2</me:bathGas> <me:experimentalRate ref1="Cl" ref2="Cl"
refReaction="R1" error= " 3.85E-12 " > 2.57E-11 </me:experimentalRate> </me:PTpair> -->
<!-- <me:PTpair units="PPCC" P=" 2.33728E+18" T="295 " precision="dd"> <me:bathGas>N2</me:bathGas> <me:experimentalRate ref1="Cl" ref2="Cl"
refReaction="R1" error= " 4.44E-12 " > 2.96E-11 </me:experimentalRate> </me:PTpair> -->
<!-- <me:PTpair units="PPCC" P=" 4.7929E+18" T="295 " precision="dd"> <me:bathGas>N2</me:bathGas> <me:experimentalRate ref1="Cl" ref2="Cl"
refReaction="R1" error= " 6.18E-12 " > 4.12E-11 </me:experimentalRate> </me:PTpair> -->
<!-- <me:PTpair units="PPCC" P=" 4.82249E+18" T="295 " precision="dd"> <me:bathGas>N2</me:bathGas> <me:experimentalRate ref1="Cl" ref2="Cl"
refReaction="R1" error= " 6.80E-12 " > 4.53E-11 </me:experimentalRate> </me:PTpair> -->

<me:PTpair units="PPCC" P=" 8.9645E+18" T="295 " precision="dd"> <me:bathGas>N2</me:bathGas> <me:experimentalRate ref1="Cl" ref2="Cl"
refReaction="R1" error= " 1.05E-11 " > 6.97E-11 </me:experimentalRate> </me:PTpair>
<me:PTpair units="PPCC" P=" 9.5858E+18" T="295 " precision="dd"> <me:bathGas>N2</me:bathGas> <me:experimentalRate ref1="Cl" ref2="Cl"
refReaction="R1" error= " 9.00E-12 " > 6.00E-11 </me:experimentalRate> </me:PTpair>
<me:PTpair units="PPCC" P=" 1.20414E+19" T="295 " precision="dd"> <me:bathGas>N2</me:bathGas> <me:experimentalRate ref1="Cl" ref2="Cl"
refReaction="R1" error= " 1.06E-11 " > 7.03E-11 </me:experimentalRate> </me:PTpair>
<me:PTpair units="PPCC" P=" 1.44379E+19" T="295 " precision="dd"> <me:bathGas>N2</me:bathGas> <me:experimentalRate ref1="Cl" ref2="Cl"
refReaction="R1" error= " 1.19E-11 " > 7.94E-11 </me:experimentalRate> </me:PTpair>
<me:PTpair units="PPCC" P=" 1.50592E+19" T="295 " precision="dd"> <me:bathGas>N2</me:bathGas> <me:experimentalRate ref1="Cl" ref2="Cl"
refReaction="R1" error= " 1.33E-11 " > 8.90E-11 </me:experimentalRate> </me:PTpair>
<me:PTpair units="PPCC" P=" 1.92604E+19" T="295 " precision="dd"> <me:bathGas>N2</me:bathGas> <me:experimentalRate ref1="Cl" ref2="Cl"
refReaction="R1" error= " 1.64E-11 " > 1.09E-10 </me:experimentalRate> </me:PTpair>
<me:PTpair units="PPCC" P=" 1.92308E+19" T="295 " precision="dd"> <me:bathGas>N2</me:bathGas> <me:experimentalRate ref1="Cl" ref2="Cl"
refReaction="R1" error= " 1.40E-11 " > 9.35E-11 </me:experimentalRate> </me:PTpair>
<me:PTpair units="PPCC" P=" 1.92604E+19" T="295 " precision="dd"> <me:bathGas>N2</me:bathGas> <me:experimentalRate ref1="Cl" ref2="Cl"
refReaction="R1" error= " 1.37E-11 " > 9.14E-11 </me:experimentalRate> </me:PTpair>
<me:PTpair units="PPCC" P=" 1.91716E+19" T="295 " precision="dd"> <me:bathGas>N2</me:bathGas> <me:experimentalRate ref1="Cl" ref2="Cl"
refReaction="R1" error= " 1.33E-11 " > 8.88E-11 </me:experimentalRate> </me:PTpair>
<me:PTpair units="PPCC" P=" 2.10355E+19" T="295 " precision="dd"> <me:bathGas>N2</me:bathGas> <me:experimentalRate ref1="Cl" ref2="Cl"
refReaction="R1" error= " 1.34E-11 " > 8.92E-11 </me:experimentalRate> </me:PTpair>
<me:PTpair units="PPCC" P=" 2.09763E+19" T="295 " precision="dd"> <me:bathGas>N2</me:bathGas> <me:experimentalRate ref1="Cl" ref2="Cl"
refReaction="R1" error= " 1.37E-11 " > 9.12E-11 </me:experimentalRate> </me:PTpair>
<me:PTpair units="PPCC" P=" 2.10059E+19" T="295 " precision="dd"> <me:bathGas>N2</me:bathGas> <me:experimentalRate ref1="Cl" ref2="Cl"
refReaction="R1" error= " 1.42E-11 " > 9.50E-11 </me:experimentalRate> </me:PTpair>
<me:PTpair units="PPCC" P=" 2.22485E+19" T="295 " precision="dd"> <me:bathGas>N2</me:bathGas> <me:experimentalRate ref1="Cl" ref2="Cl"
refReaction="R1" error= " 1.44E-11 " > 9.62E-11 </me:experimentalRate> </me:PTpair>
<me:PTpair units="PPCC" P=" 2.21893E+19" T="295 " precision="dd"> <me:bathGas>N2</me:bathGas> <me:experimentalRate ref1="Cl" ref2="Cl"
refReaction="R1" error= " 1.40E-11 " > 9.33E-11 </me:experimentalRate> </me:PTpair>
<me:PTpair units="PPCC" P=" 2.39349E+19" T="295 " precision="dd"> <me:bathGas>N2</me:bathGas> <me:experimentalRate ref1="Cl" ref2="Cl"
refReaction="R1" error= " 1.57E-11 " > 1.04E-10 </me:experimentalRate> </me:PTpair>
<me:PTpair units="PPCC" P=" 2.39941E+19" T="295 " precision="dd"> <me:bathGas>N2</me:bathGas> <me:experimentalRate ref1="Cl" ref2="Cl"
refReaction="R1" error= " 1.47E-11 " > 9.83E-11 </me:experimentalRate> </me:PTpair>
<me:PTpair units="PPCC" P=" 2.41716E+19" T="295 " precision="dd"> <me:bathGas>N2</me:bathGas> <me:experimentalRate ref1="Cl" ref2="Cl"
refReaction="R1" error= " 1.43E-11 " > 9.54E-11 </me:experimentalRate> </me:PTpair>

<!-- Knyazev -->

<!-- <me:PTpair units="PPCC" P=" 3E16" T="440" precision="dd"> <me:bathGas>He</me:bathGas> <me:experimentalRate ref1="C2H4Cl" ref2="C2H4Cl"
refReaction="R1" error= " 15 " > 77.3 </me:experimentalRate> </me:PTpair> -->
<!-- <me:PTpair units="PPCC" P=" 3E16" T="450 " precision="dd"> <me:bathGas>He</me:bathGas> <me:experimentalRate ref1="C2H4Cl" ref2="C2H4Cl"
refReaction="R1" error= " 25 " > 120.5 </me:experimentalRate> </me:PTpair> -->
<!-- <me:PTpair units="PPCC" P=" 3E16" T="450 " precision="dd"> <me:bathGas>He</me:bathGas> <me:experimentalRate ref1="C2H4Cl" ref2="C2H4Cl"
refReaction="R1" error= " 30 " > 127.4</me:experimentalRate> </me:PTpair> -->
<!-- <me:PTpair units="PPCC" P=" 3E16" T="460 " precision="dd"> <me:bathGas>He</me:bathGas> <me:experimentalRate ref1="C2H4Cl" ref2="C2H4Cl"
refReaction="R1" error= " 32 " > 157.7 </me:experimentalRate> </me:PTpair> -->
<!-- <me:PTpair units="PPCC" P=" 3E16" T="470 " precision="dd"> <me:bathGas>He</me:bathGas> <me:experimentalRate ref1="C2H4Cl" ref2="C2H4Cl"
refReaction="R1" error= " 50 " > 243.7 </me:experimentalRate> </me:PTpair> -->

```
<!-- <me:PTpair units="PPCC" P=" 3E16" T="480 " precision="dd"> <me:bathGas>He</me:bathGas> <me:experimentalRate ref1="C2H4Cl" ref2="C2H4Cl"
refReaction="R1" error= " 65 " > 321.5 </me:experimentalRate> </me:PTpair> -->
<!-- <me:PTpair units="PPCC" P=" 6E16" T="420 " precision="dd"> <me:bathGas>He</me:bathGas> <me:experimentalRate ref1="C2H4Cl" ref2="C2H4Cl"
refReaction="R1" error= " 20 " > 86.7 </me:experimentalRate> </me:PTpair> -->
<!-- <me:PTpair units="PPCC" P=" 6E16" T="430 " precision="dd"> <me:bathGas>He</me:bathGas> <me:experimentalRate ref1="C2H4Cl" ref2="C2H4Cl"
refReaction="R1" error= " 27 " > 133.6 </me:experimentalRate> </me:PTpair> -->
<!-- <me:PTpair units="PPCC" P=" 6E16" T="440 " precision="dd"> <me:bathGas>He</me:bathGas> <me:experimentalRate ref1="C2H4Cl" ref2="C2H4Cl"
refReaction="R1" error= " 40 " > 187.0 </me:experimentalRate> </me:PTpair> -->
<!-- <me:PTpair units="PPCC" P=" 6E16" T="450 " precision="dd"> <me:bathGas>He</me:bathGas> <me:experimentalRate ref1="C2H4Cl" ref2="C2H4Cl"
refReaction="R1" error= " 52 " > 250.1 </me:experimentalRate> </me:PTpair> -->
<!-- <me:PTpair units="PPCC" P=" 6E16" T="460 " precision="dd"> <me:bathGas>He</me:bathGas> <me:experimentalRate ref1="C2H4Cl" ref2="C2H4Cl"
refReaction="R1" error= " 80 " > 383.7 </me:experimentalRate> </me:PTpair> -->
<!-- <me:PTpair units="PPCC" P="12E16" T="410 " precision="dd"> <me:bathGas>He</me:bathGas> <me:experimentalRate ref1="C2H4Cl" ref2="C2H4Cl" refReaction="R1" error=
" 20 " > 99.3 </me:experimentalRate> </me:PTpair> -->
<!-- <me:PTpair units="PPCC" P=" 12E16" T="420 " precision="dd"> <me:bathGas>He</me:bathGas> <me:experimentalRate ref1="C2H4Cl" ref2="C2H4Cl"
refReaction="R1" error= " 32 " > 155.3 </me:experimentalRate> </me:PTpair> -->
<!-- <me:PTpair units="PPCC" P=" 12E16" T="430 " precision="dd"> <me:bathGas>He</me:bathGas> <me:experimentalRate ref1="C2H4Cl" ref2="C2H4Cl"
refReaction="R1" error= " 38.7" > 269.8 </me:experimentalRate> </me:PTpair> -->
<!-- <me:PTpair units="PPCC" P=" 12E16" T="430 " precision="dd"> <me:bathGas>He</me:bathGas> <me:experimentalRate ref1="C2H4Cl" ref2="C2H4Cl"
refReaction="R1" error= " 55 " > 270.4 </me:experimentalRate> </me:PTpair> -->
<!-- <me:PTpair units="PPCC" P=" 12E16" T="440 " precision="dd"> <me:bathGas>He</me:bathGas> <me:experimentalRate ref1="C2H4Cl" ref2="C2H4Cl"
refReaction="R1" error= " 80 " > 382.4 </me:experimentalRate> </me:PTpair> -->
<!-- <me:PTpair units="PPCC" P=" 18E16" T="410 " precision="dd"> <me:bathGas>He</me:bathGas> <me:experimentalRate ref1="C2H4Cl" ref2="C2H4Cl"
refReaction="R1" error= " 28 " > 136.3 </me:experimentalRate> </me:PTpair> -->
<!-- <me:PTpair units="PPCC" P=" 18E16" T="420 " precision="dd"> <me:bathGas>He</me:bathGas> <me:experimentalRate ref1="C2H4Cl" ref2="C2H4Cl"
refReaction="R1" error= " 50 " > 235.4 </me:experimentalRate> </me:PTpair> -->
<!-- <me:PTpair units="PPCC" P=" 18E16" T="430 " precision="dd"> <me:bathGas>He</me:bathGas> <me:experimentalRate ref1="C2H4Cl" ref2="C2H4Cl"
refReaction="R1" error= " 65 " > 313.9 </me:experimentalRate> </me:PTpair> -->
<!-- <me:PTpair units="PPCC" P=" 24E16" T="400 " precision="dd"> <me:bathGas>He</me:bathGas> <me:experimentalRate ref1="C2H4Cl" ref2="C2H4Cl"
refReaction="R1" error= " 25 " > 112.3 </me:experimentalRate> </me:PTpair> -->
<!-- <me:PTpair units="PPCC" P=" 24E16" T="400 " precision="dd"> <me:bathGas>He</me:bathGas> <me:experimentalRate ref1="C2H4Cl" ref2="C2H4Cl"
refReaction="R1" error= " 25 " > 110.3 </me:experimentalRate> </me:PTpair> -->
<!-- <me:PTpair units="PPCC" P=" 24E16" T="410 " precision="dd"> <me:bathGas>He</me:bathGas> <me:experimentalRate ref1="C2H4Cl" ref2="C2H4Cl"
refReaction="R1" error= " 36 " > 180.2 </me:experimentalRate> </me:PTpair> -->
<!-- <me:PTpair units="PPCC" P=" 24E16" T="420 " precision="dd"> <me:bathGas>He</me:bathGas> <me:experimentalRate ref1="C2H4Cl" ref2="C2H4Cl"
refReaction="R1" error= " 55 " > 260.7 </me:experimentalRate> </me:PTpair> -->

   </me:PTs>
  </me:conditions>

  <me:modelParameters>
   <me:grainSize units="cm-1">50.0</me:grainSize>
   <me:energyAboveTheTopHill>35.</me:energyAboveTheTopHill>
  </me:modelParameters>

  <me:control>


   <me:calcMethod xsi:type="me:marquardt">
     <me:MarquardtIterations>12</me:MarquardtIterations>
     <me:MarquardtTolerance>1e-7</me:MarquardtTolerance>
     <me:MarquardtDerivDelta>0.025</me:MarquardtDerivDelta>
   </me:calcMethod>

   <me:testDOS />
   <me:printSpeciesProfile />
   <me:testMicroRates />
   <me:testRateConstant />
   <me:printGrainDOS />
   <me:printGrainkfE />
   <me:printGrainkbE />
   <me:eigenvalues>5</me:eigenvalues>
         <me:MaximumEvolutionTime>1.e+5</me:MaximumEvolutionTime>
         <me:ForceMacroDetailedBalance />
   <me:calcMethod name="simpleCalc"/>
   <!--Use the default: simpleCalc-->
   <!-- <me:eigenvalues>5</me:eigenvalues> -->
  </me:control>

  <me:control>
   <me:calcMethod units="kJ/mol" xsi:type="me:ThermodynamicTable">
     <me:Tmin>0</me:Tmin>
     <me:Tmid>600</me:Tmid>
     <me:Tmax>1150</me:Tmax>
     <me:Tstep>25</me:Tstep>
     <me:withCellDOSCalc/>
   </me:calcMethod>
  </me:control>

</me:mesmer>
```


2. C2H4-OPT-CCSDT-madef2TZVPP

*MASS (in amu)
28.053999999999995
*END
*NUMBER OF SYMMETRY
1
*END
*FREQUENCIES (in cm-1)
823.99
863.39
936.01
1023.04
1228.83
1359.78
1467.04
1680.0
3160.63
3178.73
3258.3

3279.73
*END
*ELECTRONIC DEGENERACY
1
*END
*MOMENT OF INERTIA (in Amu.bohr**2)
73.85488044302562
61.31266127432782
12.542219168697791
*END
*LINEAR
not linear
*END
*POTENTIAL ENERGY (in hartree)
-78.50328753
*END

3. Cl-DKH-CCSDT-CBS-CCSDT-madef2TZVPP

*MASS (in amu)
35.453
*END
*ELECTRONIC DEGENERACY
2
*END
*POTENTIAL ENERGY (in hartree)
-461.1159356
*END

4. C2H3-H-Cl-TS-1-2-OPT-DKH-CCSDT-CBS-CCSDT-ma-def2-TZVPP-IRC

**POINT
*IRC
-20.0
*END
*MASS (in amu)
63.507
*END
*NUMBER OF SYMMETRY
1
*END
*FREQUENCIES (in cm-1)
196.06i
177.51
134.9
804.56

857.06
933.97
1005.98
1215.31
1365.18
1452.36
1729.43
3157.39
3178.27
3256.04
3281.46
*END
*ELECTRONIC DEGENERACY
2
*END
*MOMENT OF INERTIA (in Amu.bohr**2)
820.6120197555606
777.8299419191535
42.78207785062849
*END
*LINEAR
not linear
*END
*POTENTIAL ENERGY (in hartree)
-539.6182308460970000
*END
**END
**POINT
*IRC
-19.0
*END
*MASS (in amu)
63.507
*END
*NUMBER OF SYMMETRY
1
*END
*FREQUENCIES (in cm-1)
211.24i
181.16
140.34
802.89
854.02
931.22
1005.85
1214.84
1363.85
1451.26

1728.45
3153.44
3176.59
3255.56
3282.18
*END
*ELECTRONIC DEGENERACY
2
*END
*MOMENT OF INERTIA (in Amu.bohr**2)
812.7133075910102
770.0238305628224
42.68947708046747
*END
*LINEAR
not linear
*END
*POTENTIAL ENERGY (in hartree)
-539.6180932052220000
*END
**END
**POINT
*IRC
-18.0
*END
*MASS (in amu)
63.507
*END
*NUMBER OF SYMMETRY
1
*END
*FREQUENCIES (in cm-1)
213.25i
190.12
140.64
801.84
852.86
929.26
1001.75
1214.12
1363.77
1450.84
1727.36
3149.46
3176.52
3254.05
3281.14
*END

*ELECTRONIC DEGENERACY
2
*END
*MOMENT OF INERTIA (in Amu.bohr**2)
804.6638985622349
762.0471883177113
42.61671029370717
*END
*LINEAR
not linear
*END
*POTENTIAL ENERGY (in hartree)
-539.6179463387880000
*END
**END
**POINT
*IRC
-17.0
*END
*MASS (in amu)
63.507
*END
*NUMBER OF SYMMETRY
1
*END
*FREQUENCIES (in cm-1)
215.7i
189.5
143.97
800.12
850.57
928.48
1003.01
1212.58
1362.1
1449.92
1726.85
3143.84
3174.95
3253.06
3281.93
*END
*ELECTRONIC DEGENERACY
2
*END
*MOMENT OF INERTIA (in Amu.bohr**2)
796.8671924673363
754.3454315538416

42.52176107131958
*END
*LINEAR
not linear
*END
*POTENTIAL ENERGY (in hartree)
-539.6178204453230000
*END
**END
**POINT
*IRC
-16.0
*END
*MASS (in amu)
63.507
*END
*NUMBER OF SYMMETRY
1
*END
*FREQUENCIES (in cm-1)
225.37i
191.85
145.19
798.89
850.39
928.18
1000.25
1211.3
1360.68
1449.28
1725.44
3136.97
3175.75
3252.73
3281.06
*END
*ELECTRONIC DEGENERACY
2
*END
*MOMENT OF INERTIA (in Amu.bohr**2)
788.937341298283
746.4766970362266
42.46064426575167
*END
*LINEAR
not linear
*END
*POTENTIAL ENERGY (in hartree)

-539.6177050024000000
*END
**END
**POINT
*IRC
-15.0
*END
*MASS (in amu)
63.507
*END
*NUMBER OF SYMMETRY
1
*END
*FREQUENCIES (in cm-1)
316.23i
202.71
130.95
793.66
835.23
926.76
998.84
1207.93
1352.87
1446.51
1728.35
3127.21
3172.43
3248.89
3280.11
*END
*ELECTRONIC DEGENERACY
2
*END
*MOMENT OF INERTIA (in Amu.bohr**2)
781.2140901355308
738.8642891614144
42.34980097751887
*END
*LINEAR
not linear
*END
*POTENTIAL ENERGY (in hartree)
-539.6175335987700000
*END
**END
**POINT
*IRC
-14.0

*END
*MASS (in amu)
63.507
*END
*NUMBER OF SYMMETRY
1
*END
*FREQUENCIES (in cm-1)
239.66i
201.3
142.75
796.73
841.61
926.13
996.23
1209.17
1363.58
1447.94
1723.72
3119.45
3175.56
3250.88
3281.45
*END
*ELECTRONIC DEGENERACY
2
*END
*MOMENT OF INERTIA (in Amu.bohr**2)
773.4098648190505
731.1059063247983
42.303958499867704
*END
*LINEAR
not linear
*END
*POTENTIAL ENERGY (in hartree)
-539.6173357158880000
*END
**END
**POINT
*IRC
-13.0
*END
*MASS (in amu)
63.507
*END
*NUMBER OF SYMMETRY
1

*END
*FREQUENCIES (in cm-1)
242.32i
196.06
158.46
795.57
839.86
925.7
995.6
1206.92
1361.8
1445.87
1726.24
3106.12
3172.0
3245.86
3278.9
*END
*ELECTRONIC DEGENERACY
2
*END
*MOMENT OF INERTIA (in Amu.bohr**2)
765.7418849260448
723.5458262565899
42.19605867151836
*END
*LINEAR
not linear
*END
*POTENTIAL ENERGY (in hartree)
-539.6171436626000000
*END
**END
**POINT
*IRC
-12.0
*END
*MASS (in amu)
63.507
*END
*NUMBER OF SYMMETRY
1
*END
*FREQUENCIES (in cm-1)
250.76i
201.82
154.01
792.76

833.25
923.09
991.07
1205.94
1355.63
1444.91
1720.67
3094.79
3174.57
3248.88
3281.49
*END
*ELECTRONIC DEGENERACY
2
*END
*MOMENT OF INERTIA (in Amu.bohr**2)
758.0528083516311
715.8976525601214
42.15515579523352
*END
*LINEAR
not linear
*END
*POTENTIAL ENERGY (in hartree)
-539.6169195822840000
*END
**END
**POINT
*IRC
-11.0
*END
*MASS (in amu)
63.507
*END
*NUMBER OF SYMMETRY
1
*END
*FREQUENCIES (in cm-1)
253.94i
204.16
156.13
794.54
833.97
920.59
991.18
1204.6
1356.01
1443.31

1725.32
3076.73
3170.89
3244.75
3278.04
*END
*ELECTRONIC DEGENERACY
2
*END
*MOMENT OF INERTIA (in Amu.bohr**2)
750.473373350719
708.4253481388328
42.048025220148276
*END
*LINEAR
not linear
*END
*POTENTIAL ENERGY (in hartree)
-539.6167018409840000
*END
**END
**POINT
*IRC
-10.0
*END
*MASS (in amu)
63.507
*END
*NUMBER OF SYMMETRY
1
*END
*FREQUENCIES (in cm-1)
262.87i
209.11
159.03
789.38
828.28
918.35
985.63
1202.7
1352.81
1441.24
1719.1
3062.24
3174.33
3247.55
3281.68
*END

*ELECTRONIC DEGENERACY
2
*END
*MOMENT OF INERTIA (in Amu.bohr**2)
742.8860674378595
700.8714035355083
42.01466391887674
*END
*LINEAR
not linear
*END
*POTENTIAL ENERGY (in hartree)
-539.6164516065410000
*END
**END
**POINT
*IRC
-9.0
*END
*MASS (in amu)
63.507
*END
*NUMBER OF SYMMETRY
1
*END
*FREQUENCIES (in cm-1)
296.52i
209.62
164.16
789.23
827.56
886.37
981.5
1201.87
1349.11
1441.79
1720.18
3039.25
3169.44
3243.18
3277.71
*END
*ELECTRONIC DEGENERACY
2
*END
*MOMENT OF INERTIA (in Amu.bohr**2)
735.3993900911305
693.4925358388651

41.90685429702131
*END
*LINEAR
not linear
*END
*POTENTIAL ENERGY (in hartree)
-539.6162101861920000
*END
**END
**POINT
*IRC
-8.0
*END
*MASS (in amu)
63.507
*END
*NUMBER OF SYMMETRY
1
*END
*FREQUENCIES (in cm-1)
244.7i
160.3
159.54
761.36
782.99
888.81
979.54
1196.6
1340.94
1424.15
1713.57
3011.84
3169.0
3243.12
3271.14
*END
*ELECTRONIC DEGENERACY
2
*END
*MOMENT OF INERTIA (in Amu.bohr**2)
727.9213116239363
686.0366077597471
41.88470386672372
*END
*LINEAR
not linear
*END
*POTENTIAL ENERGY (in hartree)

-539.6159206153470000
*END
**END
**POINT
*IRC
-7.0
*END
*MASS (in amu)
63.507
*END
*NUMBER OF SYMMETRY
1
*END
*FREQUENCIES (in cm-1)
273.84i
212.44
164.24
789.67
820.56
916.56
983.39
1198.7
1349.1
1439.52
1725.05
2987.92
3165.39
3236.46
3272.22
*END
*ELECTRONIC DEGENERACY
2
*END
*MOMENT OF INERTIA (in Amu.bohr**2)
720.7477415645568
678.9823829432202
41.765358623680214
*END
*LINEAR
not linear
*END
*POTENTIAL ENERGY (in hartree)
-539.6156582429340000
*END
**END
**POINT
*IRC
-6.0

*END
*MASS (in amu)
63.507
*END
*NUMBER OF SYMMETRY
1
*END
*FREQUENCIES (in cm-1)
266.89i
214.4
165.29
792.76
822.02
921.18
985.8
1198.5
1350.38
1440.94
1735.25
2962.82
3157.04
3225.97
3262.09
*END
*ELECTRONIC DEGENERACY
2
*END
*MOMENT OF INERTIA (in Amu.bohr**2)
716.3335146796021
674.6722405983539
41.661274083703255
*END
*LINEAR
not linear
*END
*POTENTIAL ENERGY (in hartree)
-539.6154847256630000
*END
**END
**POINT
*IRC
-5.0
*END
*MASS (in amu)
63.507
*END
*NUMBER OF SYMMETRY
1

*END
*FREQUENCIES (in cm-1)
277.22i
210.26
170.3
792.48
808.18
931.07
982.92
1198.26
1343.69
1441.02
1725.76
2953.96
3130.96
3227.21
3256.53
*END
*ELECTRONIC DEGENERACY
2
*END
*MOMENT OF INERTIA (in Amu.bohr**2)
708.640156052202
666.9359654524753
41.70419060221722
*END
*LINEAR
not linear
*END
*POTENTIAL ENERGY (in hartree)
-539.6150650995380000
*END
**END
**POINT
*IRC
-4.0
*END
*MASS (in amu)
63.507
*END
*NUMBER OF SYMMETRY
1
*END
*FREQUENCIES (in cm-1)
323.4i
219.57
181.52
768.26

795.28
891.18
960.43
1181.35
1329.65
1426.56
1714.77
2780.73
3194.92
3248.26
3305.74
*END
*ELECTRONIC DEGENERACY
2
*END
*MOMENT OF INERTIA (in Amu.bohr**2)
682.0230462653238
640.498923767453
41.524122500682864
*END
*LINEAR
not linear
*END
*POTENTIAL ENERGY (in hartree)
-539.6139241550970000
*END
**END
**POINT
*IRC
-3.0
*END
*MASS (in amu)
63.507
*END
*NUMBER OF SYMMETRY
1
*END
*FREQUENCIES (in cm-1)
323.92i
209.53
218.84
761.73
763.2
900.61
952.2
1166.47
1303.83
1423.28

1699.13
2542.73
3164.23
3252.68
3267.79
*END
*ELECTRONIC DEGENERACY
2
*END
*MOMENT OF INERTIA (in Amu.bohr**2)
653.993875974922
612.9586984961152
41.03517748391211
*END
*LINEAR
not linear
*END
*POTENTIAL ENERGY (in hartree)
-539.6120293841900000
*END
**END
**POINT
*IRC
-2.0
*END
*MASS (in amu)
63.507
*END
*NUMBER OF SYMMETRY
1
*END
*FREQUENCIES (in cm-1)
230.85i
183.8
124.64
798.07
801.54
896.49
957.08
1157.79
1251.41
1414.5
1674.06
1687.08
3126.63
3174.08
3231.24
*END

*ELECTRONIC DEGENERACY
2
*END
*MOMENT OF INERTIA (in Amu.bohr**2)
639.3329836318873
598.2537168859893
41.07926675950986
*END
*LINEAR
not linear
*END
*POTENTIAL ENERGY (in hartree)
-539.6092121607780000
*END
**END
**POINT
*IRC
-1.0
*END
*MASS (in amu)
63.507
*END
*NUMBER OF SYMMETRY
1
*END
*FREQUENCIES (in cm-1)
1007.39i
125.84
254.99
454.52
788.68
806.97
844.38
925.9
1112.07
1256.25
1397.58
1675.47
3144.53
3233.84
3254.6
*END
*ELECTRONIC DEGENERACY
2
*END
*MOMENT OF INERTIA (in Amu.bohr**2)
633.8145179377376
592.152974742558

41.66154324108052
*END
*LINEAR
not linear
*END
*POTENTIAL ENERGY (in hartree)
-539.6061879538170000
*END
**END
**POINT
*IRC
0.0
*END
*MASS (in amu)
63.507
*END
*NUMBER OF SYMMETRY
1
*END
*FREQUENCIES (in cm-1)
541.45i
121.59
251.3
600.94
698.77
777.4
889.61
902.0
940.47
1216.06
1383.55
1663.86
3128.94
3239.98
3262.25
*END
*ELECTRONIC DEGENERACY
2
*END
*MOMENT OF INERTIA (in Amu.bohr**2)
652.8336154845915
609.4984075427158
43.335207943001315
*END
*LINEAR
not linear
*END
*POTENTIAL ENERGY (in hartree)

-539.6031838405310000
*END
**END
**POINT
*IRC
2.0
*END
*MASS (in amu)
63.507
*END
*NUMBER OF SYMMETRY
1
*END
*FREQUENCIES (in cm-1)
71.34i
88.41
111.12
441.59
580.35
750.71
760.7
893.14
1057.13
1383.32
1647.85
2212.53
3126.64
3225.51
3302.33
*END
*ELECTRONIC DEGENERACY
2
*END
*MOMENT OF INERTIA (in Amu.bohr**2)
692.2771293969139
646.8710368706089
45.40609255286444
*END
*LINEAR
not linear
*END
*POTENTIAL ENERGY (in hartree)
-539.6043460955130000
*END
**END
**POINT
*IRC
3.0

*END
*MASS (in amu)
63.507
*END
*NUMBER OF SYMMETRY
1
*END
*FREQUENCIES (in cm-1)
56.94i
93.33
126.1
440.53
579.97
754.76
763.93
892.1
1060.09
1382.59
1644.46
2218.45
3125.77
3228.4
3291.24
*END
*ELECTRONIC DEGENERACY
2
*END
*MOMENT OF INERTIA (in Amu.bohr**2)
693.632668993721
648.2291517542232
45.403517255997976
*END
*LINEAR
not linear
*END
*POTENTIAL ENERGY (in hartree)
-539.6043308802350000
*END
**END
**POINT
*IRC
4.0
*END
*MASS (in amu)
63.507
*END
*NUMBER OF SYMMETRY
1

*END
*FREQUENCIES (in cm-1)
66.67i
93.16
136.12
439.56
579.43
757.57
765.92
893.41
1061.59
1383.26
1646.94
2225.91
3122.58
3225.77
3285.84
*END
*ELECTRONIC DEGENERACY
2
*END
*MOMENT OF INERTIA (in Amu.bohr**2)
694.5550845726572
649.1859194865924
45.36916509704776
*END
*LINEAR
not linear
*END
*POTENTIAL ENERGY (in hartree)
-539.6043632705330000
*END
**END
**POINT
*IRC
5.0
*END
*MASS (in amu)
63.507
*END
*NUMBER OF SYMMETRY
1
*END
*FREQUENCIES (in cm-1)
66.39i
94.0
145.73
432.28

571.53
759.81
766.04
894.58
1064.24
1384.12
1650.15
2266.6
3117.44
3223.08
3280.82
*END
*ELECTRONIC DEGENERACY
2
*END
*MOMENT OF INERTIA (in Amu.bohr**2)
699.1443843076689
653.8080976435115
45.3362866663595
*END
*LINEAR
not linear
*END
*POTENTIAL ENERGY (in hartree)
-539.6044784712700000
*END
**END
**POINT
*IRC
6.0
*END
*MASS (in amu)
63.507
*END
*NUMBER OF SYMMETRY
1
*END
*FREQUENCIES (in cm-1)
68.27i
95.7
117.87
419.81
557.25
755.23
757.15
891.98
1060.99
1383.85

1651.37
2326.55
3121.36
3226.24
3290.19
*END
*ELECTRONIC DEGENERACY
2
*END
*MOMENT OF INERTIA (in Amu.bohr**2)
704.5093959431623
659.0458093123121
45.46358663184138
*END
*LINEAR
not linear
*END
*POTENTIAL ENERGY (in hartree)
-539.6046468943980000
*END
**END
**POINT
*IRC
7.0
*END
*MASS (in amu)
63.507
*END
*NUMBER OF SYMMETRY
1
*END
*FREQUENCIES (in cm-1)
73.89i
108.3
138.74
412.74
548.53
760.74
761.34
894.24
1064.29
1384.45
1647.81
2368.72
3114.54
3221.51
3274.69
*END

*ELECTRONIC DEGENERACY
2
*END
*MOMENT OF INERTIA (in Amu.bohr**2)
709.906277042436
664.2870917539927
45.619185289080484
*END
*LINEAR
not linear
*END
*POTENTIAL ENERGY (in hartree)
-539.6046760225230000
*END
**END
**POINT
*IRC
8.0
*END
*MASS (in amu)
63.507
*END
*NUMBER OF SYMMETRY
1
*END
*FREQUENCIES (in cm-1)
106.14i
60.56
71.49
399.56
533.12
742.54
746.69
890.46
1056.49
1384.5
1650.7
2422.05
3122.03
3225.49
3309.46
*END
*ELECTRONIC DEGENERACY
2
*END
*MOMENT OF INERTIA (in Amu.bohr**2)
715.0703702726379
669.3384781133442

45.73189215979964
*END
*LINEAR
not linear
*END
*POTENTIAL ENERGY (in hartree)
-539.6048961620280000
*END
**END
**POINT
*IRC
9.0
*END
*MASS (in amu)
63.507
*END
*NUMBER OF SYMMETRY
1
*END
*FREQUENCIES (in cm-1)
110.27i
67.31
90.25
398.78
532.26
748.11
752.7
890.02
1059.34
1383.36
1645.97
2429.19
3122.09
3228.2
3292.63
*END
*ELECTRONIC DEGENERACY
2
*END
*MOMENT OF INERTIA (in Amu.bohr**2)
716.6935866129882
670.893024448421
45.80056216510894
*END
*LINEAR
not linear
*END
*POTENTIAL ENERGY (in hartree)

-539.6048551845380000
*END
**END
**POINT
*IRC
10.0
*END
*MASS (in amu)
63.507
*END
*NUMBER OF SYMMETRY
1
*END
*FREQUENCIES (in cm-1)
110.09i
64.39
102.35
397.74
530.69
749.55
755.33
891.77
1059.75
1384.15
1648.69
2436.13
3118.73
3224.41
3287.84
*END
*ELECTRONIC DEGENERACY
2
*END
*MOMENT OF INERTIA (in Amu.bohr**2)
717.6367009107037
671.8384964091439
45.79820450212342
*END
*LINEAR
not linear
*END
*POTENTIAL ENERGY (in hartree)
-539.6048802042960000
*END
**END
**POINT
*IRC
11.0

*END
*MASS (in amu)
63.507
*END
*NUMBER OF SYMMETRY
1
*END
*FREQUENCIES (in cm-1)
111.33i
67.28
108.81
395.94
528.16
751.14
757.16
893.01
1060.74
1384.92
1650.51
2445.78
3116.34
3221.77
3284.5
*END
*ELECTRONIC DEGENERACY
2
*END
*MOMENT OF INERTIA (in Amu.bohr**2)
718.9317041827895
673.1211363333687
45.81056784988784
*END
*LINEAR
not linear
*END
*POTENTIAL ENERGY (in hartree)
-539.6049091201630000
*END
**END
**POINT
*IRC
12.0
*END
*MASS (in amu)
63.507
*END
*NUMBER OF SYMMETRY
1

*END
*FREQUENCIES (in cm-1)
112.06i
66.93
107.13
393.74
525.34
750.36
757.08
892.99
1060.55
1384.9
1650.28
2455.89
3116.05
3221.51
3284.55
*END
*ELECTRONIC DEGENERACY
2
*END
*MOMENT OF INERTIA (in Amu.bohr**2)
720.3240731948965
674.4791666344221
45.84490656098877
*END
*LINEAR
not linear
*END
*POTENTIAL ENERGY (in hartree)
-539.6049319083970000
*END
**END
**POINT
*IRC
13.0
*END
*MASS (in amu)
63.507
*END
*NUMBER OF SYMMETRY
1
*END
*FREQUENCIES (in cm-1)
112.43i
64.62
106.34
391.6

522.72
749.54
757.39
893.2
1060.07
1384.31
1650.66
2465.87
3115.51
3220.86
3283.9
*END
*ELECTRONIC DEGENERACY
2
*END
*MOMENT OF INERTIA (in Amu.bohr**2)
721.710789755083
675.8381211683626
45.872668587158074
*END
*LINEAR
not linear
*END
*POTENTIAL ENERGY (in hartree)
-539.6049584663810000
*END
**END
**POINT
*IRC
14.0
*END
*MASS (in amu)
63.507
*END
*NUMBER OF SYMMETRY
1
*END
*FREQUENCIES (in cm-1)
113.53i
66.69
102.46
389.42
519.9
748.8
756.87
892.86
1060.19
1384.93

1650.03
2475.57
3115.82
3221.33
3284.8
*END
*ELECTRONIC DEGENERACY
2
*END
*MOMENT OF INERTIA (in Amu.bohr**2)
723.1137003949822
677.2026829428471
45.9110174524795
*END
*LINEAR
not linear
*END
*POTENTIAL ENERGY (in hartree)
-539.6049797047860000
*END
**END
**POINT
*IRC
15.0
*END
*MASS (in amu)
63.507
*END
*NUMBER OF SYMMETRY
1
*END
*FREQUENCIES (in cm-1)
113.75i
66.0
102.21
387.34
517.16
748.76
757.3
893.13
1060.28
1384.48
1650.45
2485.12
3115.11
3220.56
3283.92
*END

*ELECTRONIC DEGENERACY
2
*END
*MOMENT OF INERTIA (in Amu.bohr**2)
724.4999074878052
678.5631841112527
45.936723376950695
*END
*LINEAR
not linear
*END
*POTENTIAL ENERGY (in hartree)
-539.6050062630850000
*END
**END
**POINT
*IRC
16.0
*END
*MASS (in amu)
63.507
*END
*NUMBER OF SYMMETRY
1
*END
*FREQUENCIES (in cm-1)
114.33i
65.46
98.15
385.21
514.55
747.45
756.78
892.83
1059.84
1384.62
1649.8
2494.42
3115.38
3220.95
3284.79
*END
*ELECTRONIC DEGENERACY
2
*END
*MOMENT OF INERTIA (in Amu.bohr**2)
725.9078216160634
679.9330623802273

45.974759236185875
*END
*LINEAR
not linear
*END
*POTENTIAL ENERGY (in hartree)
-539.6050267503600000
*END
**END
**POINT
*IRC
17.0
*END
*MASS (in amu)
63.507
*END
*NUMBER OF SYMMETRY
1
*END
*FREQUENCIES (in cm-1)
114.73i
64.39
98.02
383.18
512.1
746.82
757.21
893.09
1059.99
1385.4
1650.51
2503.58
3114.8
3220.35
3283.9
*END
*ELECTRONIC DEGENERACY
2
*END
*MOMENT OF INERTIA (in Amu.bohr**2)
727.2991232325675
681.2988727358023
46.00025049714899
*END
*LINEAR
not linear
*END
*POTENTIAL ENERGY (in hartree)

-539.6050524590630000
*END
**END
**POINT
*IRC
18.0
*END
*MASS (in amu)
63.507
*END
*NUMBER OF SYMMETRY
1
*END
*FREQUENCIES (in cm-1)
115.61i
63.92
93.79
381.1
509.36
746.44
756.7
892.76
1059.7
1385.05
1649.76
2512.5
3115.12
3220.7
3284.77
*END
*ELECTRONIC DEGENERACY
2
*END
*MOMENT OF INERTIA (in Amu.bohr**2)
728.7118546867216
682.6737289703768
46.038125716625444
*END
*LINEAR
not linear
*END
*POTENTIAL ENERGY (in hartree)
-539.6050721348120000
*END
**END
**POINT
*IRC
19.0

*END
*MASS (in amu)
63.507
*END
*NUMBER OF SYMMETRY
1
*END
*FREQUENCIES (in cm-1)
115.84i
63.97
93.77
379.11
506.56
746.51
757.16
893.04
1059.86
1385.51
1650.32
2521.28
3114.57
3220.15
3283.88
*END
*ELECTRONIC DEGENERACY
2
*END
*MOMENT OF INERTIA (in Amu.bohr**2)
730.10756753075
684.0441269490276
46.06344058209843
*END
*LINEAR
not linear
*END
*POTENTIAL ENERGY (in hartree)
-539.6050970094920000
*END
**END
**POINT
*IRC
20.0
*END
*MASS (in amu)
63.507
*END
*NUMBER OF SYMMETRY
1

*END
*FREQUENCIES (in cm-1)
116.14i
61.78
89.67
377.07
504.04
745.29
756.71
892.74
1059.29
1384.82
1649.52
2529.85
3114.7
3220.42
3284.72
*END
*ELECTRONIC DEGENERACY
2
*END
*MOMENT OF INERTIA (in Amu.bohr**2)
731.5245067625257
685.4235591002756
46.100947662617315
*END
*LINEAR
not linear
*END
*POTENTIAL ENERGY (in hartree)
-539.6051158884450000
*END
**END
